# The impact of heterogeneity on the analysis of platform trials with normally distributed outcomes

**DOI:** 10.1186/s12874-024-02293-4

**Published:** 2024-07-30

**Authors:** Kim May Lee, Richard Emsley

**Affiliations:** 1 Department of Biostatistics and Health Informatics, Institute of Psychiatry, Psychology and Neuroscience, King’s College London, 16 De Crespigny Park, SE5 8AF London, UK; 2 Department of Biostatistics and Health Informatics, Institute of Psychiatry, Psychology and Neuroscience, King’s College London, London, UK

**Keywords:** Heterogeneity, Heteroscedasticity, Non-concurrent control, Pairwise trials analysis, Platform trial designs

## Abstract

**Background:**

A platform trial approach allows adding arms to on-going trials to speed up intervention discovery programs. A control arm remains open for recruitment in a platform trial while intervention arms may be added after the onset of the study and could be terminated early for efficacy and/or futility when early stopping is allowed. The topic of utilising non-concurrent control data in the analysis of platform trials has been explored and discussed extensively. A less familiar issue is the presence of heterogeneity, which may exist for example due to modification of enrolment criteria and recruitment strategy.

**Method:**

We conduct a simulation study to explore the impact of heterogeneity on the analysis of a two-stage platform trial design. We consider heterogeneity in treatment effects and heteroscedasticity in outcome data across stages for a normally distributed endpoint. We examine the performance of some hypothesis testing procedures and modelling strategies. The use of non-concurrent control data is also considered accordingly. Alongside standard regression analysis, we examine the performance of a novel method that was known as the pairwise trials analysis. It is similar to a network meta-analysis approach but adjusts for treatment comparisons instead of individual studies using fixed effects.

**Results:**

Several testing strategies with concurrent control data seem to control the type I error rate at the required level when there is heteroscedasticity in outcome data across stages and/or a random cohort effect. The main parameter of treatment effects in some analysis models correspond to overall treatment effects weighted by stage wise sample sizes; while others correspond to the effect observed within a single stage. The characteristics of the estimates are not affected significantly by the presence of a random cohort effect and/ or heteroscedasticity.

**Conclusion:**

In view of heterogeneity in treatment effect across stages, the specification of null hypotheses in platform trials may need to be more subtle. We suggest employing testing procedure of adaptive design as opposed to testing the statistics from regression models; comparing the estimates from the pairwise trials analysis method and the regression model with interaction terms may indicate if heterogeneity is negligible.

**Supplementary Information:**

The online version contains supplementary material available at 10.1186/s12874-024-02293-4.

## Introduction

A platform trial approach can speed up intervention discovery programs. This is achieved by allowing adding arms and other pre-specified design adaptations during the lifetime of a study. The approach can be considered as an extension of a multi-arm multi-stage adaptive design: A control arm is always active for recruitment and new arms can be added after the onset of a platform trial, while other pre-specified adaptations may be employed as in multi-arm multi-stage designs. The time to discover interventions and the number of patients involved in clinical studies can be reduced by such an approach when compared to conducting multiple independent two-arm studies. This is because a platform trial is set-up once for the lifetime of a study, and there is only one control arm being used to evaluate multiple intervention simultaneously [1, 2]. Examples of platform trials include STAMPEDE for prostate cancers [3], REMAP-CAP for community-acquired Pneumonia [4] FLAIR for chronic lymphocytic leukaemia [5], and RECOVERY for confirmed or suspected Covid-19 [6].

For interventions that were added after the onset of a platform trial, the comparisons with the treatment of the control arm can either utilise all the control data that are available for the analysis, or only the data of those controls who were randomized during the same period as the subjects who were randomized to the intervention arms. The later control data are often known as contemporaneous or concurrent control data. To our knowledge, the methodology research for the analysis of platform trials has been focusing on this aspect [7–13]. From the statistical point of view, the choice between these two analysis strategies is related to the bias-variance tradeoff when there is a time trend in the study. The former strategy may lead to a biased estimated treatment difference that has a smaller variance than the latter strategy when there is a time trend in the trial. Several researchers identified that using a categorical time modelling approach fitted to all data can give valid treatment effect estimates when the outcomes of all active arms (within a stage/period) are influenced by a time trend in a similar manner, even if the trend is non-linear in recruitment time [8, 10]. From the implementation perspective, some stakeholders were concerned about the temporality of the nonconcurrent control and bias introduced by different confounders related to time; but in some trial scenarios where recruitment is challenging, the use of nonconcurrent control data may be justified [14, 15].

Here we focus on another issue in platform (and adaptive) trials that is less familiar to the trial community, namely heterogeneity across stages of a study. More specifically we refer to the scenario where the responses of experimental subjects who were recruited to one trial stage are consistently different to the responses of subjects who were recruited to another stage. This phenomenon may cause the following issues in a platform trial with a continuous endpoint: i) the group average effect of a treatment (either of an intervention or the control arm) becomes inconsistent across stages, and/or ii) variances of outcome becomes inconsistent across stages, i.e., heteroscedasticity in outcome data. Example of reasons include modification of eligibility criteria as arms are dropped or added [16]; there exist patients who respond differently to the same treatment across stages in the presence/absence of other intervention arms (an effect that is analogous to the placebo effect) [17]; and/or recruitment started with a limited number of trial sites/ regions but was broadened to include more sites/ regions as the trial progresses [18].

In fact, the topic of heterogeneity has been widely explored in the context of meta-analysis where the results of multiple studies are combined for making an overall inference, see for example the discussion between clinical heterogeneity and statistical heterogeneity [19]. In recent years, heterogeneity in treatment effects in fixed trials has drawn the attentions of trial statisticians due to the Covid-19 pandemic outbreak [20–22]. To mimic the scenario where there are multiple independent studies, it has been suggested to split the data of a single trial into separated distinct blocks/stages, e.g., before pandemic, during pandemic and after pandemic, such that the methods in the topics of meta-analysis may be applied analogously [20, 21].

Methods to assess heterogeneity in treatment effects across periods in either a fixed trial or an adaptive trial include: Cochran’s Q test that was first proposed for meta-analysis [20, 21], a multi-step testing procedure for heterogeneity in adaptive designs [23] and the test for qualitative interaction between stages of adaptive designs [24]. Nevertheless, some may argue that the data of fixed trials and adaptive trials are unlikely to be large enough to conduct such a test with high power at the commonly considered significance level of 5% [23, 25].

To account for heterogeneity in treatment effects across stages in the analysis of fixed trials, some researchers proposed to utilise the approaches for meta-analysis [20], Bayesian hierarchical methods [21] or machine learning techniques [22]. We are not aware of any proposal/ investigation that accounts for heterogeneity in the analysis of platform trials; the closest one is the proposal of a network meta-analysis framework that assesses the impact of utilising non-concurrent controls on the analysis of platform trials [9].

Our goal here is to explore the impact of heterogeneity on the analysis of a platform trial by a simulation study. Many have suggested that heterogeneity across trial stages would cause complications in result interpretations [16, 18, 26, 27]. They have described the issue to raise awareness of the problem but to our knowledge, nobody has explored numerically how the presence of heterogeneity affects the inference. We examine the performance of several hypothesis testing procedures and modelling approaches for a two-stage design that adds an arm in the second stage. In addition to the usual regression modelling approaches, we consider the application of a novel modelling approach, known as pairwise trials analysis method, which was proposed for the analysis of an innovative (non-adaptive) design [28]. This model includes fixed effects for making multiple research comparisons in the analysis, as opposed to the fixed effects for independent studies in the context of network meta-analysis.

The structure of this article is as follows. We first review some testing and estimation strategies for the analysis of platform trials. We then describe our simulation study and selected analysis strategies in more details. We explore multiple scenarios to examine the impact of heterogeneity on the analysis. Finally we discuss the simulation results and comment on the analysis strategy for platform trials that have a more complex design than the considered setting.

## Methods

Focusing on many-to-one comparisons, we are interested in making inference about each research comparison in terms of hypothesis test and estimation of treatment effect. Hereafter we refer to a research comparison as the comparison between an intervention and the control treatment. We refer to a treatment effect as the mean difference between the responses of an intervention and the responses of the control treatment. We describe some options of analysis strategy for hypothesis test and estimation in this section.

Consider a two-stage design that started with three arms in stage one; a forth arm is added to the second stage of the study. Let $y_{ijk}$ be the response of experimental subject *i* in arm *j* of stage *k*, with $i=1,..., n_{jk}, j=A, B, C,$ for intervention arms and $j=0$ for control arm, $k=1,2,$ indicates the trial stage, and $n_{jk}$ is the sample size of arm *j* in stage *k*. Let $n_j$ be the total sample size of arm *j* in the study with $n_{j1}+n_{j2}=n_j$. Consider stage one has three arms $j=0, A, B,$ and stage two has four arms $j=0, A, B, C,$ where intervention *C* is the newly added arm with $n_{C1}=0$.

In the scenario where both the effect of a treatment and the outcome are homogeneous across stages, a normally distributed outcome of arm *j* has mean $\mu '_{j}$ and variance $\sigma ^2$, i.e., $y_{ijk}\sim N(\mu '_{j}, \sigma ^2)$. (Otherwise we have $y_{ij1} \sim N(\mu _{j1}, \sigma ^2 )$ for stage one responses and $y_{ij2} \sim N(\mu _{j2}, \sigma ^2 + \sigma _e^2 )$ for stage two responses as considered in our simulation study in “Simulation study” section).

### Hypothesis testing procedures

For each research comparison, consider the one-sided null hypothesis $H_{0j'}: \mu '_{j}- \mu '_{0} = 0$ where a positive difference represents a treatment benefit, $j'=A, B, C$.

One testing strategy that ignores the fact that arm *C* was added to stage two of the study is to apply a simple *T*-test to each null hypothesis. For $H_{0C}$, one may use all the control data or only the concurrent control data, i.e., stage two control data, to conduct the test as described in the Background section.

Without making adjustment for testing more than one hypothesis in a study, one may apply the simple *T*-test to each null hypothesis at $\alpha$ significance level. Alternatively one may apply a procedure/ correction to control the familywise type I error rate (FWER), i.e., the probability of rejecting at least one null hypothesis, at the desired level. For example, to apply Bonferroni correction in our context, a simple *T*-test is applied to each hypothesis at $\alpha /3$ significance level in order to ensure that the FWER is achieved at $\alpha$ level.

A less conservative strategy than the *T*-test with Bonferroni correction is to consider Dunnett test in platform trials; the Dunnett test accounts for the correlation between the test statistics of the research comparisons due to utilising a common control group [29].

To ensure the FWER is controlled in the strong sense, i.e., FWER is guaranteed for any configuration of true and non-true null hypotheses (whether the global null hypothesis is true or not), we can apply a closed testing procedure with a test [30, 31]. More specifically, this involves testing intersection hypotheses in addition to the usual elementary/individual $H_{0j'}$. The closed test principle states that an individual null hypothesis is only rejected when the elementary hypothesis and all the associated intersection hypotheses are also rejected at $\alpha$-level. An intersection hypothesis can be tested using testing procedure such as the Dunnett test, Simes test, Sidak test and the likelihood ratio tests [31]. Table 1 shows the rejection set of hypotheses when the closed test principle is applied to the considered trial design here.

**Table 1:** Closed testing procedure for three research comparisons: reject a hypothesis when both the intersection hypothesis and the rejection set are each rejected by a testing procedure at significant level $\alpha$

Intersection hypothesis	Rejection set
$$^* H_{0A} \cap H_{0B} \cap H_{0C}$$	
$H_{0A} \cap H_{0B}$	$H_{0A} \cap H_{0B} \cap H_{0C}$
$^* H_{0A} \cap H_{0C}$	$H_{0A} \cap H_{0B} \cap H_{0C}$
$^* H_{0B} \cap H_{0C}$	$H_{0A} \cap H_{0B} \cap H_{0C}$
$H_{0A}$	$H_{0A} \cap H_{0B} \cap H_{0C}$ and $H_{0A} \cap H_{0B}$ and $H_{0A} \cap H_{0C}$
$H_{0B}$	$H_{0A} \cap H_{0B} \cap H_{0C}$ and $H_{0A} \cap H_{0B}$ and $H_{0B} \cap H_{0C}$
$H_{0C}$	$H_{0A} \cap H_{0B} \cap H_{0C}$ and $H_{0A} \cap H_{0C}$ and $H_{0B} \cap H_{0C}$

^*^When computing the stage one *p*-value for the combination test, these are equivalent to the corresponding hypothesis without $H_{0C}$

Note that the above described testing strategies have not accounted for the adaptation made to the study, i.e., adding arm *C* to the second stage of the study. In other words, these strategies ignore the two-stage design structure and any dissimilarities between the stages. Testing strategies that account for design modifications include the combination test procedures [32] and the procedure based on the conditional error principle [33]. These strategies allow the application of pre-specified design modifications without compromising the trial integrity, in the sense that the type I error rate or the FWER is controlled at the desired level. The conditional error principle was also proposed to enable modification of (fixed) trials that were affected by the Covid-19 pandemic [21].

The idea of the conditional error principle is as follows. First, compute the conditional probability of rejecting a null hypothesis, conditioned on the data prior to any design modification, i.e., stage one data in our context. This conditional error rate is then used as the error rate for evaluating the remaining hypotheses based on the data post-modification, i.e., stage two data in our context. Proceeding in such a way maintains the error rate at the desired level.

On the other hand, combination test procedures work by combining the stage-wise *p*-values via a pre-specified function and comparing the resulting value with a significant level for a decision to reject or not reject a null hypothesis [34]. More specifically, the data of each stage are used to compute a respective *p*-value, e.g., based on a *T*-test or a Dunnett test. The resulting stage-wise *p*-values are then combined, e.g., by multiplication (for the Fisher’s combination method) or weighted summation (for the Inverse Normal method).

One may also apply the closed test principle when employing a combination test procedure or the conditional error principle. This means the intersection hypotheses are tested analogously. For intersection hypotheses that involve the newly added research comparison *C*, we don’t have any information about it when computing the corresponding (stage one) conditional error rate or *p*-value; the data of arm *C* will only play a role in the second stage computation [7, 31]. For example, the intersection $^* H_{0A} \cap H_{0B} \cap H_{0C}$ is equivalent to $^* H_{0A} \cap H_{0B}$ for stage one computation.

### Estimation of treatment effects

One simple way to estimate a treatment effect of a research comparison is to consider the difference between the sample mean response of an intervention arm and the sample mean response of the control arm. Together with their standard deviations, *T*-statistic can be constructed for the corresponding hypothesis test.

Alternatively one may fit a regression model to all data, providing the summary statistics of the least squared estimates of treatment effects. In the context of a two-stage design that adds an arm, one may add a stage effect to the regression model to reflect the two-stage design structure. Such modelling approaches use all control data in the estimation of all treatment effects, including that of research comparison *C*. Some authors find that the latter strategy provides a valid inference when there is a non-differential time trend in the study, i.e., the data of different arms are affected by a trend in a similar way that the trend is offset in the estimated treatment effects [8, 10]. To account for a differential trend, including a treatment-by-stage interaction term in the regression model may give valid inference but at the cost of not utilising the non-concurrent control data for research comparison *C* [10].

A pairwise trials analysis method has been proposed for an innovative design (known as Practical design) where there are subgroups of subjects defined by randomization lists; each list contains a subset of interventions that a patient is eligible for randomization [28]. This modelling approach fits a single model to duplicated data that are involved in each pairwise comparison, and adjusts for the pairwise comparisons with fixed effects. The standard errors of the estimated treatment effects are then computed using a sandwich variance to reflect the dependency between the duplicated data. (See example in the next section.)

We propose a version of the pairwise trials analysis approach for the analysis of platform trials, especially when there are many arms added at various stages and some patients are eligible for randomization to some arms but not all (scenario not considered here). Our context here is analogous to the Practical design [28] when view the design as recruitment of two different subgroups and ignore the stage/ recruitment period. More specifically, patients in stage one are ‘eligible’ for randomization to the control arm, arm *A*, and *B*; patient in stage two are ‘eligible’ for randomization to the control arm, arm *A*, *B* and *C*. Such a design has four arms and some patients are ineligible for randomization to arm *C*.

### Simulation study

We now describe the setting of our simulation study and some analysis strategies in more details. The purpose of the simulation study is to explore the impact of heterogeneity on the inference when some analysis strategies are employed. More specifically, we consider the mean and variance of $y_{ijk}$ by stages and vary their values in the underlying data generating mechanism. We then apply some of the analysis strategies to the simulated data and examine the properties of the inference.

We follow some of the numerical setting of a previous work [7]: Consider each arm has a total sample size of $n_j=120$ experimental subjects, which would give a power of 0.9 to detect an effect size of 0.38 between an intervention arm (*A* or *B*) and the control arm, at a significant level of $\alpha =5\%$ based on a one-sided *T*-test. For the added arm *C*, set $n_{c2}=n_c=120$; this means control data that are concurrent to arm *C* will be less than $n_{c2}$.

Three respective timings of adding an arm are considered. More specifically, arm *C* is added after 25%, 50% and 75% of $n_j$ subjects are recruited to the study, for $j=0, A, B$. For each experimental subject, a two-step randomization procedure is applied: subjects are first randomized to a two-arm cohort, followed by randomization to an arm within the cohort. More specifically, a cohort consists of one arm for an intervention, another for the control treatment. For example, cohort *A* consists of those in arm *A* and the control arm; cohort *B* consists of those in arm *B* and the control arm; cohort *C* consists of those in arm *C* and the control arm. Table 2 shows the randomization ratios for the trial settings considered in our simulation. We use simple randomization with the considered ratios for each step of the procedure in our simulation.

Let $\mathbf {Y_{1} }= (\textbf{y}_{0, 1}, \textbf{y}_{A,1}, \textbf{y}_{B,1})$ be the stage one outcome data and $\mathbf {Y_{2} }= (\textbf{y}_{0, 2}, \textbf{y}_{A,2}, \textbf{y}_{B,2}, \textbf{y}_{C,2})$ be the stage two outcome data, where $\textbf{y}_{jk}$ is the vector of responses of arm *j* in stage *k*. We simulate $\mathbf {Y_{1}}$ from a multivariate normal distribution with mean $\mu _1={( \mu _{01} \textbf{1}_{n_{01}}^T , \mu _{A1}\textbf{1}_{n_{A1}}^T , \mu _{B1}\textbf{1}_{n_{B1}}^T )}^T$ and covariance $\Sigma _1= \sigma ^2 \textbf{I}_{n1} + \sigma ^2_c \textbf{1}_{n1} \textbf{1}_{n1}^T$ where $\textbf{1}_{r}^T$ is a $(r \times 1)$ vector of ones, $\textbf{I}_{r}$ is a $(r \times r)$ identity matrix, $\sigma ^2_c$ is a random cohort effect and $\sigma ^2=1$, following the setting of [7]. For stage two data, we simulate $\mathbf {Y_{2}}$ from another multivariate normal distribution with mean $\mu _2={( \mu _{02} \textbf{1}_{n_{02}}^T , \mu _{A2}\textbf{1}_{n_{A2}}^T , \mu _{B2}\textbf{1}_{n_{B2}}^T, \mu _{C2}\textbf{1}_{n_{C2}}^T )}^T + \tau$ and covariance $\Sigma _2= (\sigma ^2 +\sigma ^2_e) \textbf{I}_{n2} + \sigma ^2_c \textbf{1}_{n2} \textbf{1}_{n2}^T$ where $\tau$ is a fixed cohort effect/ time trend and $\sigma ^2_e$ is the extra variability in stage two responses.

**Table 2:** Data generating mechanisms for our simulation study. All scenarios are considered with setting one for each set of $\{\sigma _c, \sigma _e\}$ and $\tau =0$ and $\tau =-0.5$ respectively. Scenario SA.b are considered for setting two and three respectively for each set of $\{\sigma _c, \sigma _e\}$ and $\tau =0$ and $\tau =-0.5$ respectively

	Stage one	Stage two
Setting one: add an arm at midpoint		
Stage wise sample size:	$n_{01}=n_{A1}=n_{B1}=60$, $n_{C1}=0$	$n_{02}=n_{A2}=n_{B2}=60$, $n_{C2}=120$
Randomization ratios:		
Step one	90:90 for cohort *A* : *B*	80:80:140 for cohort *A* : *B* : *C*
Step two	60:30 for arm *A* : control	60:20 for arm *A* : control
	60:30 for arm *B* : control	60:20 for arm *B* : control
		120:20 for arm *C* : control
Setting two: add an arm after 25% recruitment		
Stage wise sample size:	$n_{01}=n_{A1}=n_{B1}=30$, $n_{C1}=0$	$n_{02}=n_{A2}=n_{B2}=90$, $n_{C2}=120$
Randomization ratios:		
Step one	45:45 for cohort *A* : *B*	120:120:150 for cohort *A* : *B* : *C*
Step two	30:15 for arm *A* : control	90:30 for arm *A* : control
	30:15 for arm *B* : control	90:30 for arm *B* : control
		120:30 for arm *C* : control
Setting three: add an arm after 75% recruitment		
Stage wise sample size:	$n_{01}=n_{A1}=n_{B1}=90$, $n_{C1}=0$	$n_{02}=n_{A2}=n_{B2}=30$, $n_{C2}=120$
Randomization ratios:		
Step one	135:135 for cohort *A* : *B*	40:40:130 for cohort *A* : *B* : *C*
Step two	90:45 for arm *A* : control	30:10 for arm *A* : control
	90:45 for arm *B* : control	30:10 for arm *B* : control
		120:10 for arm *C* : control
Scenarios	$\{\mu _{01}, \mu _{A1}, \mu _{B1} \}$	$\{\mu _{02}, \mu _{A2}, \mu _{B2}, \mu _{C2} \}$
S0: Null scenario	$\{0, 0, 0\}$	$\{0, 0, 0, 0\}$
SC.m: Moderate heterogeneity in control arm	$\{0, 0, 0\}$	$\{0.2, 0, 0, 0\}$
SC.b: Big heterogeneity in control arm	$\{0, 0, 0\}$	$\{0.7, 0, 0, 0\}$
SA.m: Moderate heterogeneity in arm *A*	$\{0, 0, 0\}$	$\{0, 0.2, 0, 0\}$
SA.b: Big heterogeneity in arm *A*	$\{0, 0, 0\}$	$\{0, 0.7, 0, 0\}$

#### Data generating mechanisms

We consider the following data generating mechanisms when $\tau =0$ and $\tau \ne 0$ respectively:

- Scenario 1 has $$\{\sigma _c, \sigma _e\}= \{0, 0\}$$
- Scenario 2 has $$\{\sigma _c, \sigma _e\}= \{0.38, 0\}$$
- Scenario 3 has $$\{\sigma _c, \sigma _e\}= \{0, 0.38\}$$
- Scenario 4 has $$\{\sigma _c, \sigma _e\}= \{0.38, 0.38\}$$

When $\tau =0$, it is the scenario that has no time trend or fixed cohort effect, otherwise $\tau \ne 0$. Non-zero $\sigma _c$ corresponds to having a random cohort effect while non-zero $\sigma _e$ means stage one and stage two outcome data have different variability, i.e., an example of heteroscedasticity.

In addition to the null hypothesis scenario, denoted by S0, where $\mu _1=\mu _2=\textbf{0}$, we set $\mu _{02} > 0$ and $\mu _{A2} > 0$ respectively in the simulation to explore the impact of heterogeneity in treatment effect across stages. The case with $\mu _{02} > 0$ corresponds to the scenario that all research comparisons in stage two are no longer under the null hypothesis setting; while the case with $\mu _{0A} > 0$ corresponds to only research comparison *A* in stage two is no longer under the null. We consider moderate and large heterogeneous effects respectively. Table 2 shows the numerical values of $\mu _1$ and $\mu _2$ for the scenarios considered in our simulation study.

#### Estimands

We are interested in making comparisons between each intervention arm and the control arm. We want to identify if each of the null hypotheses, $H_{0A}$ , $H_{0B}$ , $H_{0C}$, can be rejected when a testing procedure is applied. We also want the respective estimated treatment effects when an estimation strategy is employed.

#### Competing analysis strategies

We examine the following testing strategies:

- Apply the simple *T*-test to each null hypothesis at $\alpha =5\%$. We denote these by *T-test A*, *T-test B*, and, *T-test C* for research comparisons *A*, *B*, *C*, respectively using concurrent control data, and by *T-test C-nc* for research comparison *C* that employs all the stage one and two control data in the computation.
- Apply the simple *T*-test with Bonferroni correction to test the three hypotheses; control the FWER at 5%. We denote this by *Bonferroni* and *Bonferroni-nc* for computations that use the concurrent control data only and that with the combined control data, respectively.
- Apply Dunnett test followed by the closed testing procedure to control the FWER at 5%. We denote this strategy by *Dunnett.closed* and *Dunnett.closed-nc* for computations that use the concurrent control data only and that with the combined control data, respectively. This approach ignores the two stage design. Table 1 shows the intersection hypotheses of the closed testing procedure.
- Apply Fisher’s combination test followed by the closed testing procedure to control the FWER at 5%. We denote this strategy by *Combination.closed*. Let $p_1$ and $p_2$ denote the stage one and stage two *p*-values of a hypothesis. The Fisher’s combination test proceeds as follows: reject a null hypothesis when $p_1 p_2 \le exp [ -1/2 \chi ^2_4(1-\alpha )]$ where $\chi ^2_4(1-\alpha )$ is the $(1-\alpha )$-quantile of the central $\chi ^2$ distribution with 4 degrees of freedom [34]. The closed testing principle states that a null hypothesis is rejected only when both the elementary and intersection hypotheses are rejected. We compute the stage-wise *p*-values from a Dunnett test for all the elementary and intersection hypotheses. For stage one intersection hypotheses that involve the new research comparison *C*, they are equivalent to the corresponding intersection/elementary hypotheses without $H_{0C}$. For example, $H_{0A} \cap H_{0B} \cap H_{0C}= H_{0A} \cap H_{0B}$ in stage one; the same stage one *p*-value is used for these two intersection hypotheses.

For the estimation of treatment effects, we compare the following modelling approaches:

- Simple linear regression model ignoring the two-stage design structure. We denote this by *M0*. The fitted model is
  $$\begin{aligned} E({y}_{ijk} )= \hat{\beta _0} + \hat{\theta }_A \ I(j=A) + \hat{\theta }_B \ I(j=B) + \hat{\theta }_C \ I(j=C) \end{aligned}$$
  where $I(\cdot )$ is an indicator function and $E({y}_{ijk})$ denotes the fitted value. Here $\hat{\theta }_j$ is interpreted as the estimated treatment effect for the comparison between intervention *j* with the control treatment, where $j=A, B, C$.
- Linear regression model adjusting for a stage effect. We denote this by *M.stage*. The fitted model is
  $$\begin{aligned} E({y}_{ijk} )= \hat{\beta _0} + \hat{\theta }_A \ I(j=A) + \hat{\theta }_B \ I(j=B) + \hat{\theta }_C \ I(j=C) + \hat{\tau } \ I(k=2) \end{aligned}$$
  where $\hat{\tau }$ is the estimated fixed cohort effect/ trend. This model tends to provide a valid inference when there is a non-differential time trend across stages [8, 10].
- Linear regression model adjusting for a stage effect and an interaction term for stage-by-intervention *A*. We denote this by *M.stageXA*. The fitted model is
  $$\begin{aligned} E({y}_{ijk} ) = & \hat{\beta _0} + \hat{\theta }_A \ I(j=A) + \hat{\theta }_B \ I(j=B) + \hat{\theta }_C \ I(j=C) \\ & + \hat{\tau } \ I(k=2) + \hat{\eta }_A \ I(j=A) \ I(k=2) \end{aligned}$$
  where $\hat{\eta }_A$ represents the additional time effect on arm *A* in stage two, relative to the time trend on other arms in stage two. In other words, this model accounts for a differential time effect on arm *A* while other arms are affected by a trend equally [10].
- Linear regression model adjusting for a stage effect and two interaction terms for i) stage-by-intervention *A* and ii) stage-by-intervention *B* respectively. We denote this by *M.stageXAXB*. The fitted model is
  $$\begin{aligned} E({y}_{ijk}) = & \hat{\beta _0} + \hat{\theta }_A \ I(j=A) + \hat{\theta }_B \ I(j=B) + \hat{\theta }_C \ I(j=C) \\ & + \hat{\tau } \ I(k=2) + \hat{\eta }_A \ I(j=A) \ I(k=2) + \hat{\eta }_B \ I(j=B) \ I(k=2) \end{aligned}$$
  where the additional model parameter, $\hat{\eta }_B$, represents the additional time effect on arm B in stage two, relative to the time trend on the control arm in stage two.
- The pairwise trials analysis method [28]. We denote this by *M.new*. Prior to fitting a model, we prepare the data as follows: For stage one data, create two copies of the original control data: one set is used for making the inference about research comparison *A*, another for research comparison *B*. Likewise for stage two data, create three copies of the original control data that are to be used for making the inference about each of the three research comparisons. Then create a dummy variable $Z_{ijk}$ to indicate the research comparisons. For example, the responses of arm *A* and of a copy of all the original control data have $Z_{ijk}=A_0$, the responses of arm *B* and of a copy of all the original control data have $Z_{ijk}=B_0$, and the responses of arm *C* and of a copy of the original stage two control data have $Z_{ijk}=C_0$. A model is then fitted to the data, resulting in
  $$\begin{aligned} E({y}_{ijk} )= & \hat{\beta _0} + \hat{\theta }_A \ I(j=A) + \hat{\theta }_B \ I(j=B) + \hat{\theta }_C \ I(j=C) \\ & + \gamma _{B0} \ I(Z_{ijk} =B_0)+ \gamma _{C0} \ I(Z_{ijk} =C_0) \end{aligned}$$
  where $\gamma _{B0}$ and $\gamma _{C0}$ represent the comparisons. The label of the data entries is used as the “clustering” variable for computing the sandwich variance.

Note that we use $\hat{\theta }_j, j=A, B, C,$ for the estimates of treatment effects in all these competing strategies as $\hat{\theta }_j$ is the parameter of interests in the main model.

#### Performance measures

For hypothesis testing, we focus on the probability of rejecting a hypothesis or at least one hypothesis that make comparison between an intervention arm and the control arm (depending on the testing strategy).

For estimation, we consider the distribution of the sample estimates, $\hat{\theta }_j$ , from the analysis models. We present box plot to examine the mean, median and variability of the sample estimates. We also present the plot of root mean squared error (rMSE) of the estimates.

## Results

We repeat the randomization and the respective data generating process 5000 times and summarise the analysis results across the replications. All simulation are implemented on *R 4.2.2*.

**Fig. 1:**
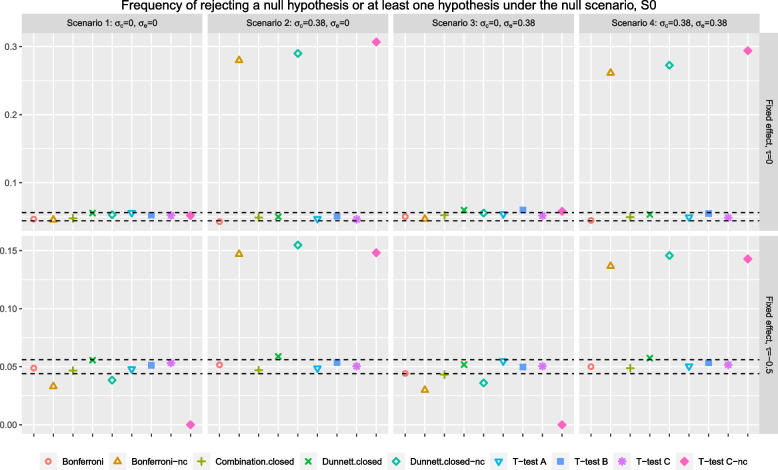
For *T-test A*, *T-test B*, *T-test C* and *T-test C-nc*, the points indicate the probability of rejecting a null hypothesis. For *Bonferroni* , *Bonferroni-nc*, *Combination.closed*, *Dunnett.closed*, *Dunnett.closed-nc*, the points indicate the FWER. Arm *C* is added after recruitment of 50% of $n_j$, $j=0, A, B,$ in the simulation

### Hypothesis testing

Figure 1 shows the rejection probability of an individual null hypothesis for *T-test A*, *T-test B*, *T-test C* and *T-test C-nc*, and the rejection probability of at least one null hypothesis for the rest of the competing procedures, when arm *C* is added after recruitment of 50% of $n_j$, $j=0, A, B$. The dashed lines correspond to the 95% simulation error coverage for the nominal rate of 5%. The first row of plots are the results for trial settings where there are no fixed cohort effect in the data while the second row for the settings with a fixed cohort effect of -0.5. Each column corresponds to a scenario with $\{\sigma _c, \sigma _e\}$.

In general, we find that all the considered testing procedures that do not utilise the stage one control data for research comparison *C* tend to control the FWER or the type one error rate well. We see that some points are slightly outside of the 95% simulation error coverage, which might be due to the small number of trial replications considered in the simulation.

When there are no fixed and random cohort effects in the data, i.e., Fig. 1 top row of plots scenarios 1 and 3, the procedures that utilise stage one control data for research comparison *C* seem to control the associated error rate well even when stage two data have a larger variability than stage one data. When there is a random cohort effect (and no fixed cohort effect) in the data, i.e., Fig. 1 top row of plots scenarios 2 and 4, these procedures lead to an inflation of the associated error rate, which can be as high as 30% (as opposed to the nominal level of 5%). This is because for research comparison *C*, the pooled estimate of variance in these procedures underestimates the true value, leading to more rejections of the null hypothesis [7]. We see that *Bonferroni-nc* and *Dunnett.closed-nc* have a slightly smaller inflation than *T-test C-nc* in these scenarios. This might be because these procedures focus on FWER and that the pooled estimates of variances for the other two research comparisons are over-estimating the true value [7], which lead to fewer rejections of the corresponding hypotheses; so the overall rejection rate of at least one null hypothesis is slightly lower than the type one error rate of *T-test C-nc*.

**Fig. 2:**
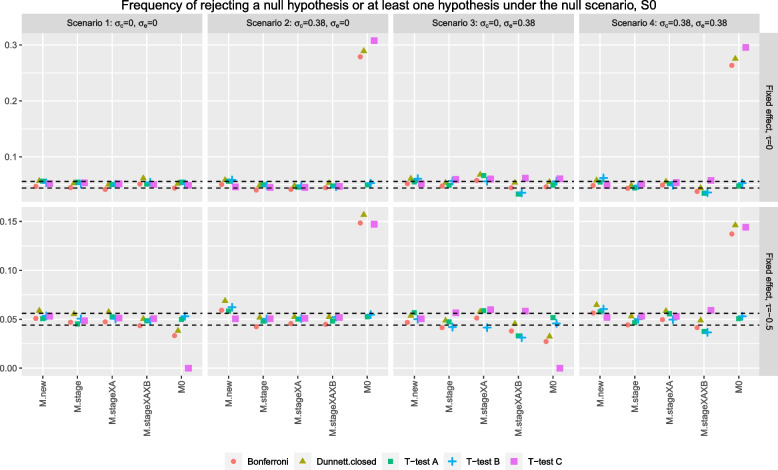
The points denoted by Bonferroni and Dunnett.closed correspond to the FWER. The points denoted by *T-test A*, *T-test B*, and *T-test C* correspond to the type one error rate

For settings where a fixed and a random cohort effect are present in the data, i.e., Fig. 1 bottom row of plots scenarios 2 and 4, the procedures that utilise the combined control data for research comparison *C* lead to an inflation of error rate, which can be as high as 15%; but these magnitudes are slightly smaller than the settings that do not have a fixed cohort effect (but a random cohort effect). This might be explained by the fact that i) the pooled estimates of variances are biased (for all research comparisons) and ii) there is a negative fixed cohort effect in the combined control data. When there is a positive fixed cohort effect (simulation result not presented here), these procedures can lead to an inflation of the associated error rate where the magnitudes are larger than the settings that do not have a fixed cohort effect (but a random cohort effect). The magnitude of inflation depends on the values of $\tau >0$.

For settings where there is a fixed cohort effect but not a random cohort effect in the data, i.e., Fig. 1 bottom row of plots scenarios 1 and 3, the procedures that utilise the combined control data for research comparison *C* lead to a deflation of the associated error rate, with *T-test C-nc* having no rejection of the null hypothesis at all. This can be explained by the fact that the pooled estimate of treatment effect is bias negatively in the presence of a negative fixed cohort effect; the rejection frequency of *Bonferroni-nc* and *Dunnett.closed-nc* are due to the rejection of the other two null hypotheses. When there was a positive fixed cohort effect, we observed an inflation of error rate, which magnitude depends on the values of $\tau >0$ (simulation result not presented here).

Figure 2 shows the rejection probability under the same trial scenarios to those considered in Fig. 1 but computed using the test statistics of an analysis model (indicated on the *x*-axis). More specifically, the test statistics of a fitted model is used to test the null hypotheses by i) rejecting each null hypothesis at $\alpha =5\%$ level, denoted by *T-test A*, *T-test B*, and *T-test C*, respectively; ii) rejecting each null hypothesis at $\alpha /3$ level, denoted by *Bonferroni* or iii) a Dunnett test followed by a closed testing procedured, denoted by *Dunnett.closed*. The results from using the test statistics of model *M0* are analogous to the results of *T-test C-nc*, *Bonferroni-nc* and *Dunnett.closed-nc* in Fig. 1 as the least square estimates from *M0* are equivalent to the sample mean differences. For hypothesis tests based on the test statistics of other modelling approaches, the corresponding error rate may not be controlled at level $\alpha$ when the underlying model assumptions fail to hold, e.g., some of the rejection probabilities are outside of the 95% simulation error coverage for scenarios 2,3 and 4. This in contrast with the results on Fig. 1 shows that the non-model based hypothesis testing strategies are more robust when heterogeneity presents in the data.

### Estimation of treatment effects

We refer to sample estimates as the estimated treatment effect of a single research comparison obtained from the simulation replications.

For figures that display the box plots of sample estimates: each row and column correspond to a research comparison and a scenario with different $\{\sigma _c, \sigma _c\}$. From all box plots, we see that the sample estimates from all competing strategies are normally distributed and the mean and median are closed to each other. Comparing the box plots of an approach across the columns, i.e., scenarios with different $\{\sigma _c, \sigma _c\}$, we see that the properties of $\hat{\theta }_j$ are consistent except the samples from *M0*.

First focus on the scenarios with $\tau =0$ and the setting where arm *C* is added after recruitment of 50% of $n_j$, $j=0, A, B,$ in the simulation.

**Fig. 3:**
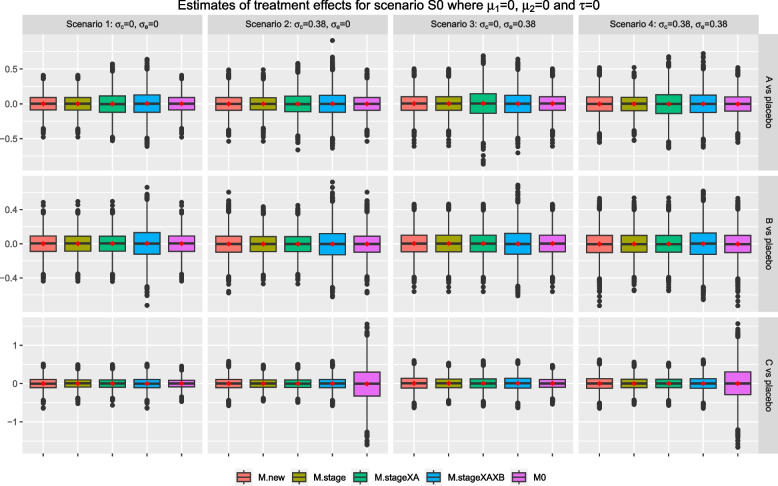
Box plots of sample estimates for each research comparison for scenario S0 with $\tau =0, \mu _1=0, \mu _2=0$. Arm *C* is added after recruitment of 50% of $n_j$, $j=0, A, B$

Under the null scenario, S0, where all research comparisons have zero treatment effects across the stages, Fig. 3 shows that the sample estimates from all methods have a mean and median that are close to zero for all research comparisons. For research comparison *B*, the sample estimates from *M.stageXAXB* have the largest interquartile range when compared with other methods across the scenarios. This observation is similar for research comparison *A* when we have scenarios 1 and 2; for the other two scenarios, the sample estimates from *M.stageXA* have the largest interquartile range. These indicate that including intervention-by-stage interaction term(s) in the analysis model increases the spread of the sample estimates of treatment effects; it is also indicated by the longer tails in the corresponding box plots. For research comparison *C*, we see that the sample estimates from *M0* have the smallest interquartile range and the shortest tails for scenarios 1 and 3; but the opposite characteristics were observed for scenarios 2 and 4. The later might be due to the fact the *M0* does not account for the extra variability and the random cohort effect in the data.

We now consider scenarios SC.m and SC.b where the control treatment has a null effect in stage one but $\mu _{02}>0$; while the mean responses of all interventions are zero in both stages. When $\mu _{02}=0.2$ in scenario SC.m, the treatment effect weighted by stage wise sample sizes is equal to 0 x 0.5 + (-0.2) x 0.5= -0.1 for both research comparisons *A* and *B*. When $\mu _{02}=0.7$ in scenario SC.b, the treatment effect weighted by stage wise sample sizes is equal to 0 x 0.5 + (-0.7) x 0.5= -0.35 for both research comparisons *A* and *B*. For research comparison *C*, $\mu _{0C}- \mu _{02}=0-0.2=-0.2$ for scenario SC.m and $\mu _{0C}- \mu _{02}=0-0.7=-0.7$ for scenario SC.b, respectively.

**Fig. 4:**
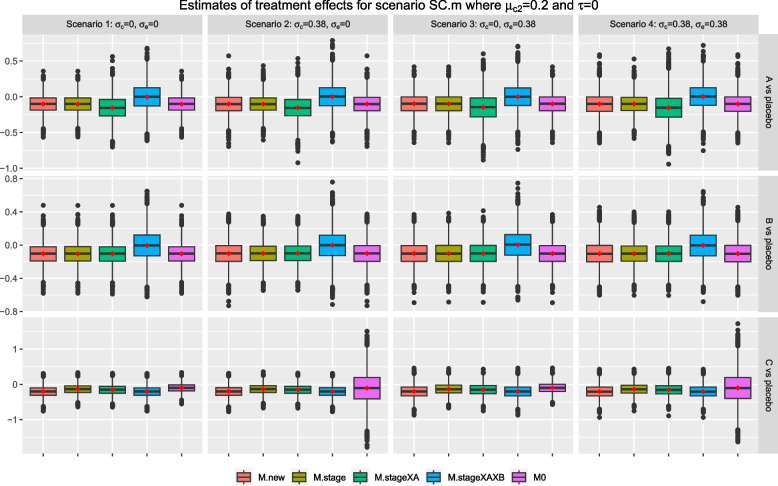
Box plots of sample estimates for each research comparison for scenario SC.m with $\tau =0, \mu _{02}=0.2$ and other stage wise means are equal to 0. Arm *C* is added after recruitment of 50% of $n_j$, $j=0, A, B$

**Fig. 5:**
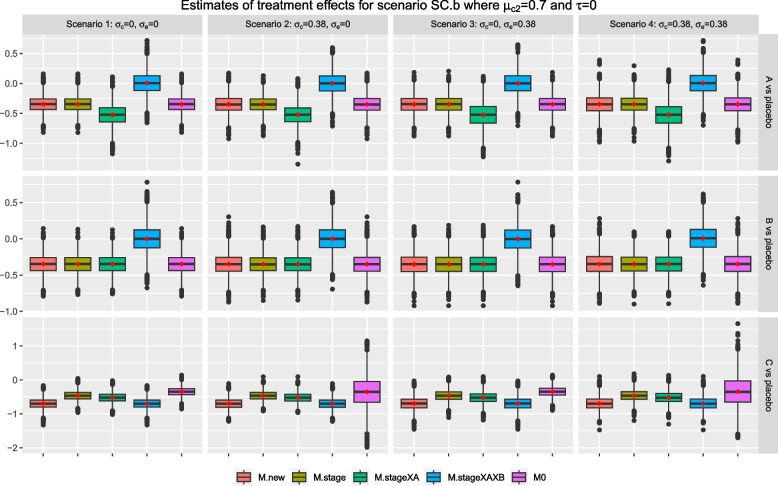
Box plots of sample estimates for each research comparison for scenario SC.b with $\tau =0, \mu _{02}=0.7$ and other stage wise means are equal to 0. Arm *C* is added after recruitment of 50% of $n_j$, $j=0, A, B$

For research comparison *A*, Figs. 4 and 5 show that the mean and median of the sample estimates from *M.new*, *M.stage*, and *M0* are closed to the corresponding weighted treatment effects; while those from *M.stageXAXB* are closed to $\mu _{01}=0$; but those from *M.stageXA* are further away from zero and the weighted treatment effects. A similar finding is observed for research comparison *B* except that *M.stageXA* is now giving a mean and median that are closed to the corresponding weighted treatment effects. For research comparison *C*, *M.new*, and *M.stageXAXB* give sample estimates that have a mean and median that are closed to the true treatment effect of stage two; while the mean and median of sample estimates from all other methods are consistently larger than the true treatment effect and closer to 0 across scenarios 1-4 in the figures. As in scenario S0, Fig. 3, the sample estimates from *M0* for research comparison *C* have the shortest tails in scenarios 1 and 3, and the longest tails in scenarios 2 and 4.

**Fig. 6:**
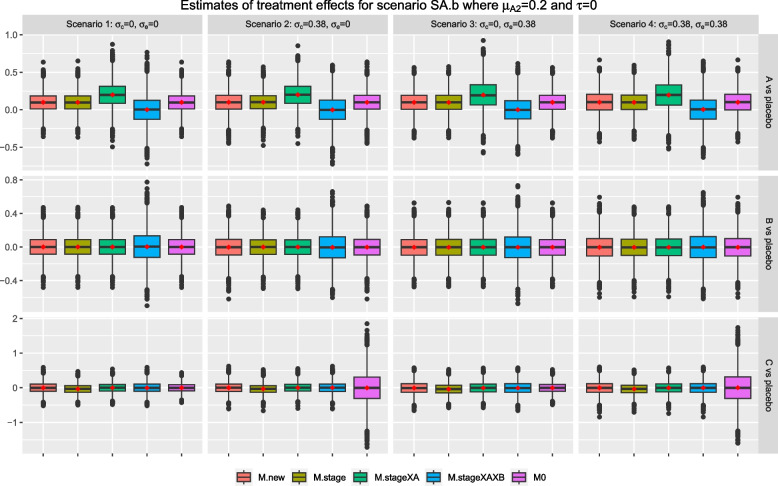
Box plots of sample estimates for each research comparison for scenario SA.m with $\tau =0, \mu _{A2}=0.2$ and other stage wise means are equal to 0. Arm *C* is added after recruitment of 50% of $n_j$, $j=0, A, B$

**Fig. 7:**
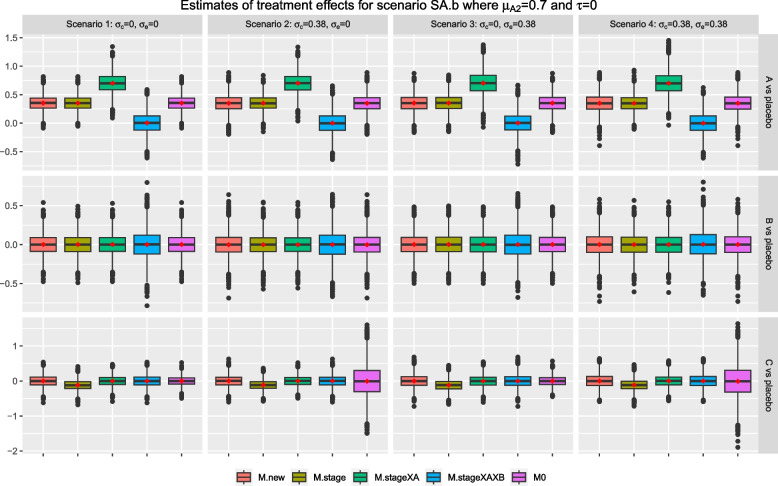
Box plots of sample estimates for each research comparison for scenario SA.b with $\tau =0, \mu _{A2}=0.7$ and other stage wise means are equal to 0. Arm *C* is added after recruitment of 50% of $n_j$, $j=0, A, B$

Consider scenarios SA.m and SA.b where intervention *A* has a null effect in stage one but $\mu _{A2}>0$; while all other arms have a null effect in both stages. Figures 6 and 7 show that the characteristics of the sample estimates from all methods for research comparison *B* remain the same as in the null scenario, S0, in Fig. 3 as expected. For research comparison *C*, the pattern of observations is similar to the scenario S0 except for *M.stage* where the resulting mean and median are consistently smaller than the true value of zero across the scenarios. This observation is more obvious for scenario SA.b in Fig. 7 where $\mu _{A2}=0.7$ than scenario SA.m where $\mu _{A2}=0.2$.

For research comparison *A*, Figs. 6 and 7 show that the sample estimates from *M.stageXAXB* have a mean and median that are closed to zero, i.e., the true value of stage one effect, $\mu _{A1}$. The sample estimates from *M.stageXA* have a mean and median that are closed to the true value of the second stage effect, $\mu _{A2}$. The other methods give sample estimates that have a mean and median that are closed to the weighted value of the stage-wise effects, weighted by the proportion of stage-wise sample size. For example in scenario SA.m, the weighted value is 0 x 0.5 +0.2 x 0.5=0.1 while in scenario SA.b, the weighted value is 0 x 0.5 + 0.7 x 0.5 = 0.35.

**Fig. 8:**
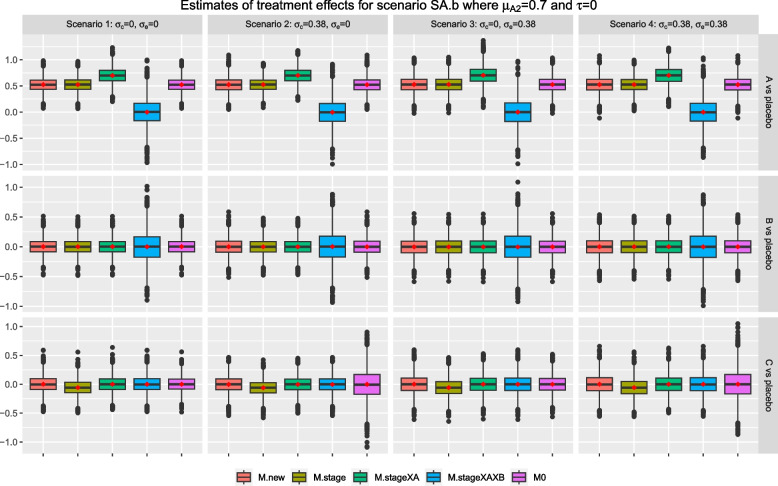
Box plots of sample estimates for each research comparison for scenario SA.b with $\tau =0, \mu _{A2}=0.7$ and other stage wise means are equal to 0. Arm *C* is added after recruitment of 25% of $n_j$, $j=0, A, B$

**Fig. 9:**
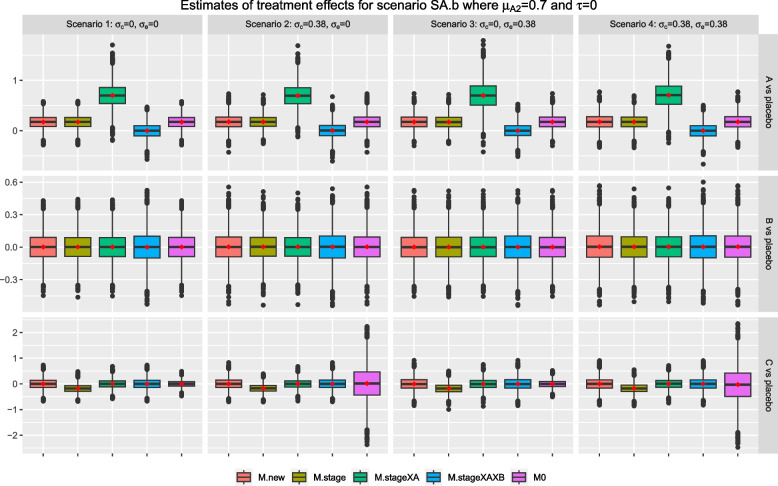
Box plots of sample estimates for each research comparisons for scenario SA.b with $\tau =0$. Arm *C* is added after recruitment of 75% of $n_j$, $j=0, A, B$

Figures 8 and 9 show the results for scenario SA.b when arm *C* is added after recruitment of 25% and 75% of $n_j$ respectively, for $j=0, A, B$. The findings are similar to the setting when arm *C* is added after recruitment of 50% of $n_j$. The mean of treatment effects for research comparison *A* from methods *M.new*, *M.stage* and *M0* are closed to the weighted treatment effects of 0 x 0.25 + 0.7 x 0.75=0.525 and 0 x 0.75 + 0.7 x 0.25=0.175 respectively. For research comparison *C*, we also see that the mean and median of sample estimates from *M.stage* are consistently smaller than the true value in these two settings.

When a fixed cohort effect of $\tau =-0.5$ exists in the data, all observations about research comparisons *A*, *B* and *C* are similar, except the sample estimates of research comparison *C* from *M0*. The latter is not surprising as model *M0* does not account for the fixed cohort effect in the data and that all the control data are used in the analysis of research comparison *C*. The corresponding plots can be found in the Supplementary document Figures 1-5.

**Fig. 10:**
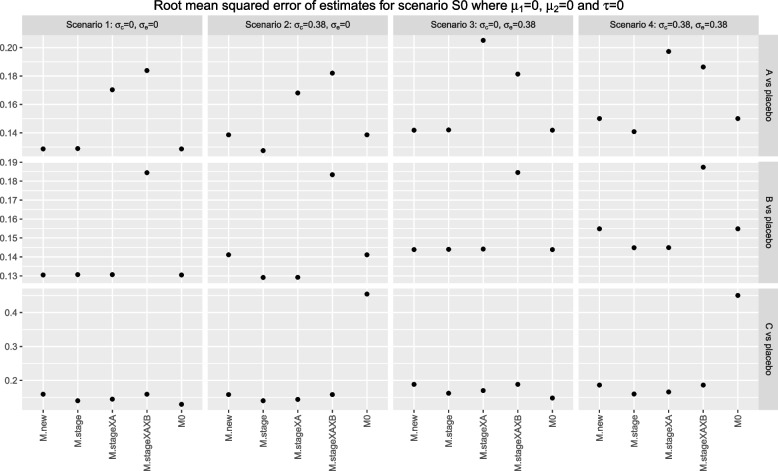
Root mean squared error of sample estimates for each research comparison for scenario S0. Arm *C* is added after recruitment of 50% of $n_j$, $j=0, A, B$

We now consider the rMSE of the estimates from different analysis strategies. Figure 10 shows the rMSE for scenario S0 with $\tau =0$. For research comparisons *A*, estimates from *M*.*stageXAXB* have the highest rMSE for settings where $\sigma _e=0$, i.e., scenarios 1 and 2, followed by the estimates from *M*.*stageXA*. The opposite is observed for settings where $\sigma _e=0.38$, i.e., scenarios 3 and 4. The rMSE of estimates from *M*.*stage* is the lowest across all scenarios 1-4 while those from *M*.*new* and *M*0 are comparable to each other.

For research comparison *B*, estimates from *M*.*stageXAXB* have the highest rMSE for all scenarios 1-4. For settings where $\sigma _c=0.38$, i.e., scenarios 2 and 4, the estimates from *M*.*new* and *M*0 have a slightly larger magnitude of rMSE than those from *M*.*stage* and *M*.*stageXA*. The estimates from these four analysis approaches have similar magnitudes of rMSE for settings where $\sigma _c=0$, i.e., scenarios 1 and 3.

For research comparison *C*, estimates from *M*0 have the highest rMSE when $\sigma _c=0.38$, i.e., scenarios 2 and 4. Apart from these, the rMSE from all methods are comparable to each other within each scenario.

These patterns of finding are similar for different trial settings. The corresponding plots of the rMSE are presented in Figures 6-14 in the Supplementary document, for scenarios S0, SC.m, SC.b, SA.m and SA.b under setting one with $\tau =0$ and $\tau =-0.5$, respectively.

**Table 3:** Summary of the performance of hypothesis testing procedures

Is the error rate being controlled at the nominal level?		
	Utilise concurrent control data$^\mathrm{a}$	Utilise all control data$^\mathrm{a}$
Scenario 1: $\sigma _c=0, \sigma _e=0$	Yes	Yes/No$^\mathrm{b}$
Scenario 2: $\sigma _c=0.38, \sigma _e=0$	Yes	No
Scenario 3: $\sigma _c=0, \sigma _e=0.38$	Yes	Yes/No$^\mathrm{b}$
Scenario 4: $\sigma _c=0.38, \sigma _e=0.38$	Yes	No

$^\mathrm{a}$In the test of research comparison *C*

$^\mathrm{b}$Yes, when there is no fixed cohort effect. No, when there is a fixed cohort effect

**Table 4:** Summary of the properties of estimated treatment effects from different analysis approaches. WTE= estimated treatment effect weighted by the stagewise sample size, S1= stage one treatment effect, S2= stage two treatment effect

	Stage two control arm has a positive mean (SC.m and SC.b)			Stage two arm A has a positive mean (SA.m and SA.b)		
Research comparison	$A^\mathrm{a}$	$B^\mathrm{b}$	$C^\mathrm{c}$	$A^\mathrm{a}$	$B^\mathrm{b}$	$C^\mathrm{c}$
*M.new*	WTE	WTE	Unbiased	WTE	Unbiased	Unbiased
*M.stage*	WTE	WTE	Biased	WTE	Unbiased	Biased
*M.stageXA*	Biased	WTE	Biased	S2	Unbiased	Unbiased
*M.stageXAXB*	S1	S1	Unbiased	S1	Unbiased	Unbiased
*M0*	WTE	WTE	Biased	WTE	Unbiased	Unbiased

$^\mathrm{a}$*M.stageXAXB* and *M.stageXA* give the largest (or second largest) rMSE

$^\mathrm{b}$*M.stageXAXB* gives the largest rMSE

$^\mathrm{c}$*M0* sometimes gives the largest rMSE, otherwise all five methods are comparable

## Discussion

In this article we consider the issue of heterogeneous treatment effects and heteroscedasticity in outcomes across stages of platform trials. We examine the methods for hypothesis testing and estimation, respectively. For the latter, we evaluate the novel method that was known as the pairwise trials analysis, alongside other regression analyses. We note that some of the scenarios/settings that we explored here are similar to those explored for utilising non-concurrent control data. More specifically, the presence of a fixed cohort effect is analogous to the scenario where there is a step time trend [8, 10] while the presence of heterogeneity in treatment effect across stages is analogous to the scenario where there is a differential time trend in the data [9].

Table 3 shows a summary of the performance of testing procedures in terms of controlling error rate. In the presence of heteroscedasticity in outcome data and/or a random cohort effect, our simulation findings show that the hypothesis testing strategies using only the concurrent control data are more robust than those utilising non-concurrent control data. The comparison between Figs. 1 and 2 indicates that the testing results from a testing procedure can be different to the testing result following from a modelling strategy. Moreover, the modelling approaches that utilise non-concurrent control data may not provide reliable estimates under scenarios with heterogeneity. For example, the estimated treatment effects for research comparison *C* from *M0* are highly variable when there is a random cohort effect, while those from *M.stage* are biased when heterogeneity in treatment effects exists. These findings have not been emphasized by existing work that focused on the utility of non-concurrent control data, yet it was suggested to employ *M.stage* in the presence of non-differential time trend.

We do not present the hypothesis testing results when there is heterogeneity in the effect of an arm across stages. This is because in such scenarios, the true underlying hypothesis is changing from one stage to another. For the same reason, we do not focus on the power of the design, i.e., the probability of rejecting the hypothesis(es) in this work. We see from the estimation results that different modelling approaches give different ‘type’ of estimated effects when we focus on the main parameter of treatment effects, i.e., $\hat{\theta }_j$, in the models. Nevertheless, we see that the properties of $\hat{\theta }_j$ from each competing strategy are consistent under scenarios with different values of $\{\sigma _c, \sigma _e\}$ except those from *M0*. Table 4 summarises the properties of the estimated treatment effects from different analysis approaches.

For the initial research comparisons *A* and *B* respectively, we find that $\hat{\theta }_j$ from the regression model adjusting for all possible intervention-by-stages interaction terms, i.e. *M.stageXAXB*, correspond to the treatment effects of stage one when heterogeneity in treatment effect across stages exists. The $\hat{\theta }_A$ from *M.stageXA* corresponds to stage two treatment effect of research comparison *A* under scenarios with $\mu _{A2}>0$, otherwise it is bias under scenarios with $\mu _{C2}>0$. Moreover, the estimates from these two models have relatively larger rMSE than those from other analysis approaches. On the other hand, $\hat{\theta }_j$ from the pairwise trials analysis method, *M0*, and *M.stage* correspond to weighted estimated treatment effects, for $j=A, B$. When an unequal ratio is used to randomize patients into arms, we find that the $\hat{\theta }_j$ from the pairwise trials analysis and *M0* are equivalent to $w_{j1} \bar{X}_{j1}+w_{j2}\bar{X}_{j2} - (w_{01} \bar{X}_{01}+w_{02}\bar{X}_{02})$ where $w_{jk}$ and $\bar{X}_{jk}$ correspond to $n_{jk}/n_j$ and sample mean of arm *j* in stage *k* respectively.; this is not the case for $\hat{\theta }_j$ from *M.stage*.

For research comparison *C*, we find that $\hat{\theta }_C$ from *M.stageXAXB* and the pairwise trials analysis are unbiased for all the considered scenarios, including the scenarios with a fixed cohort effect. In the absence of heterogeneity in treatment effects, the estimates from all methods have a mean and median that are of similar magnitudes, but the estimated samples from *M.stageXAXB* tend to have a higher variability than those from the other approaches. Note that in our investigation arm *C* only exists in stage two. If there was another stage such that there is heterogeneity of its effect across stages two and three, then the findings will be similar to those of comparison *A* under scenarios SA.m and SA.b when utilising concurrent control data. For approaches that utilise non-concurrent control, the finding will be analogous to the differential time trend explored by [9].

Based on the observations from our simulation study, we suggest to apply both a regression model with all the potential intervention-by-stages interaction terms and the pairwise trials analysis method to the analysis of platform trials that add arms. The model of the pairwise trials analysis method can be extended to include important covariates in the models [28].

We considered some not-too-small values for $\mu _{02}>0$ and $\mu _{A2}>0$ respectively in the simulation. When these parameters have small values, e.g., $\mu _{02}=0.02$ and $\mu _{A2}=0.02$ in the respective simulation, we see that the impact of heterogeneity is negligible (results not presented here). In practice, the true data generating mechanism is unknown. Comparing the estimated treatment effects from the pairwise trials analysis method and the model that adjusted for interactions terms may indicate if heterogeneity is negligible.

Like other work that is based on simulation, we presented the findings of a limited number of scenarios/ settings. For example, we did not consider the scenarios where the size of the control arm in stage two is updated to match the size of the newly added arm. We hypothesise that the results summarized in Tables 3 and 4 remain valid under this setting. We also did not consider other types of outcomes such as binary and survival endpoints. We think the impact of heterogeneity on these outcomes is similar to our findings here. For platform trials with repeated measurements, future work can extend our investigation to explore the impact of heterogeneity.

Futhermore, we have explored a not-so-complex design in the presence of heterogeneity: two stages and adding one arm is the only adaptation. For hypothesis testing, we note that the conditional error principle and the combination test methods can be applied analogously to designs that have more than two stages and those include other design adaptations [35, 36].

For platform trials that only allow for adding arms in multiple stages, the pairwise trials analysis method and the model adjusting for all possible intervention-by-stage interaction terms may also be applied analogously; even though the latter would then involve more parameters for intervention-by-stage interactions. Another scenario where the latter may not give estimates of good property is the scenario where some patients are eligible for randomization to some arms but not all. One may explore this by considering the Practical design [28] in the platform trial setting.

For platform trial designs that include other type of adaptations, such as covariate adaptive randomization, response adaptive randomization and dropping arms, more investigation on the topic of estimation is required as each adaptation may cause a different type of bias in the estimated treatment effects when the analysis model does not account for it, see, for example [31, 36, 37]. Future work may explore how the estimated treatment effects from the pairwise trials analysis may be extended to account for the bias due to the implementation of other design adaptations.

We do not comment on selecting a testing procedure and whether multiplicity correction shall be applied to platform trials as the decision is dependent on trial settings [15]. Interested readers are referred to [17] and the reference therein.

## Conclusion

In view of heterogeneity in treatment effect across stages, the specification of null hypotheses in platform trials may need to be more subtle. Moreover, we suggest conducting hypothesis test following the procedure considered during the design stage of the study, while the modelling strategies provide estimated treatment effects adjusted for other prognostic covariates.

In addition to exploring stage-wise heterogeneity by presenting key patient characteristics and results by independent stages and treatment groups [16], we suggest to implement the pairwise trials analysis method and the modelling approach with interaction terms respectively. The differences between the estimated treatment effects from these two modelling strategies may indicate if heterogeneity is negligible when present. We agree with others that trial data is unlikely to detect heterogeneity with high power when a formal hypothesis test is conducted [25]. Non-concurrent control data may be considered for trial settings where the impact of heterogeneity is small and recruitment is challenging.

### Supplementary Information

Supplementary Material 1.

## Data Availability

No datasets were generated or analysed during the current study.

## Abbreviation

FWER: Familywise type I error rate
