# Supplementary material for: The impact of heterogeneity on the analysis of platform trials with normally distributed outcomes
Source: BMC Med Res Methodol. 2024 Jul 30;24:163. doi: 10.1186/s12874-024-02293-4 (PMC11290279; doi:10.1186/s12874-024-02293-4)
Supplement: Supplementary file 1 — Supplementary Material 1. [file 12874_2024_2293_MOESM1_ESM.zip › 12874_2024_2293_MOESM1_ESM/Heterogeneity_in_platform_trials_supplementary document.pdf]

1 Supplementary material

Figures 1-5 show the box plots of sample estimates for scenarios SC.m, SC.b, SA.m and SA.b under setting one with $\tau = -0.5$.

Figures 6-14 show the root mean squared error of the estimates for scenarios S0, SC.m, SC.b, SA.m and SA.b under setting one with $\tau = 0$ and $\tau = -0.5$, respectively.

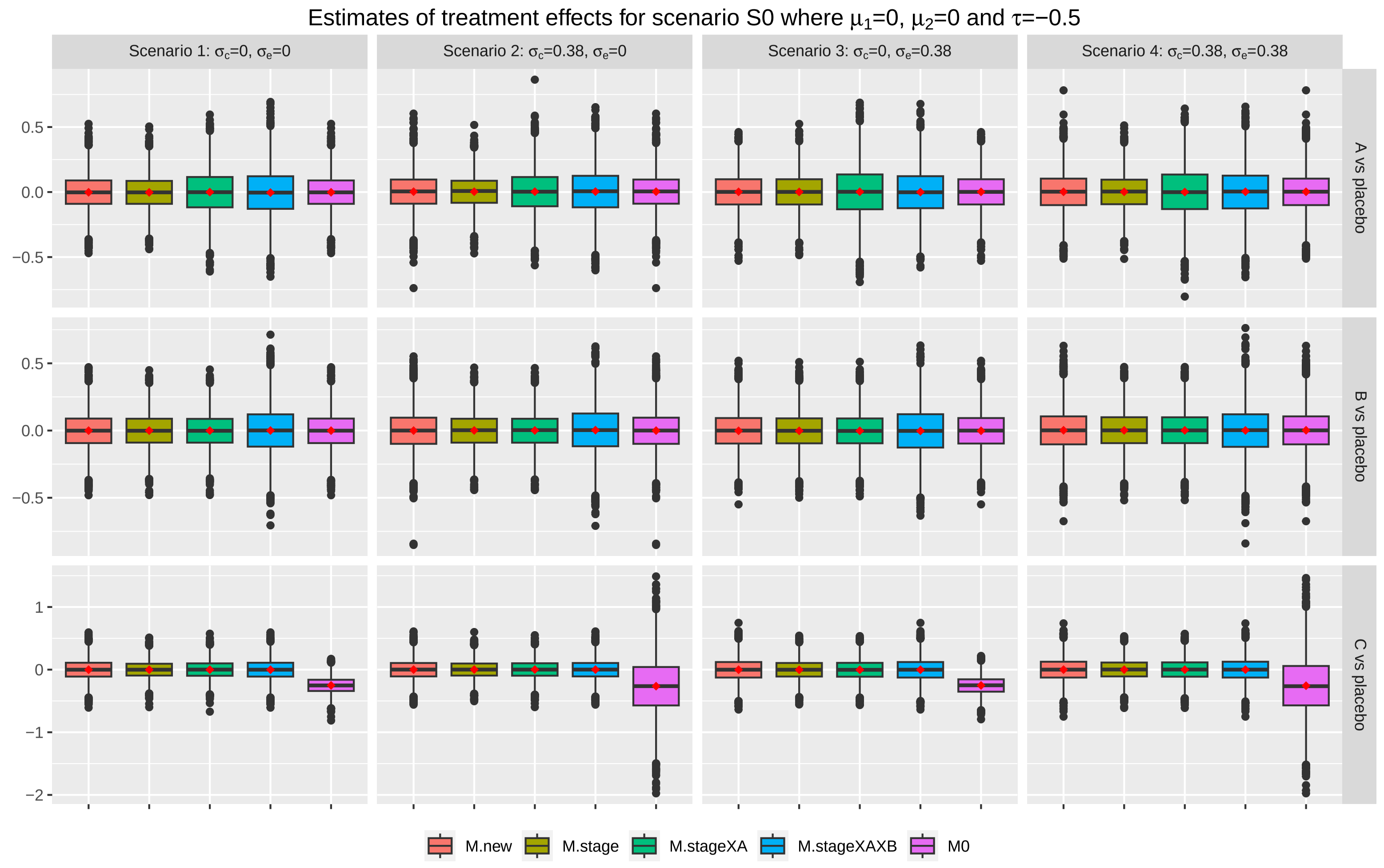

Figure 1: Box plots of sample estimates for each research comparison for scenario S0. Arm C is added after recruitment of 50% of n_j, $j = 0, A, B$.

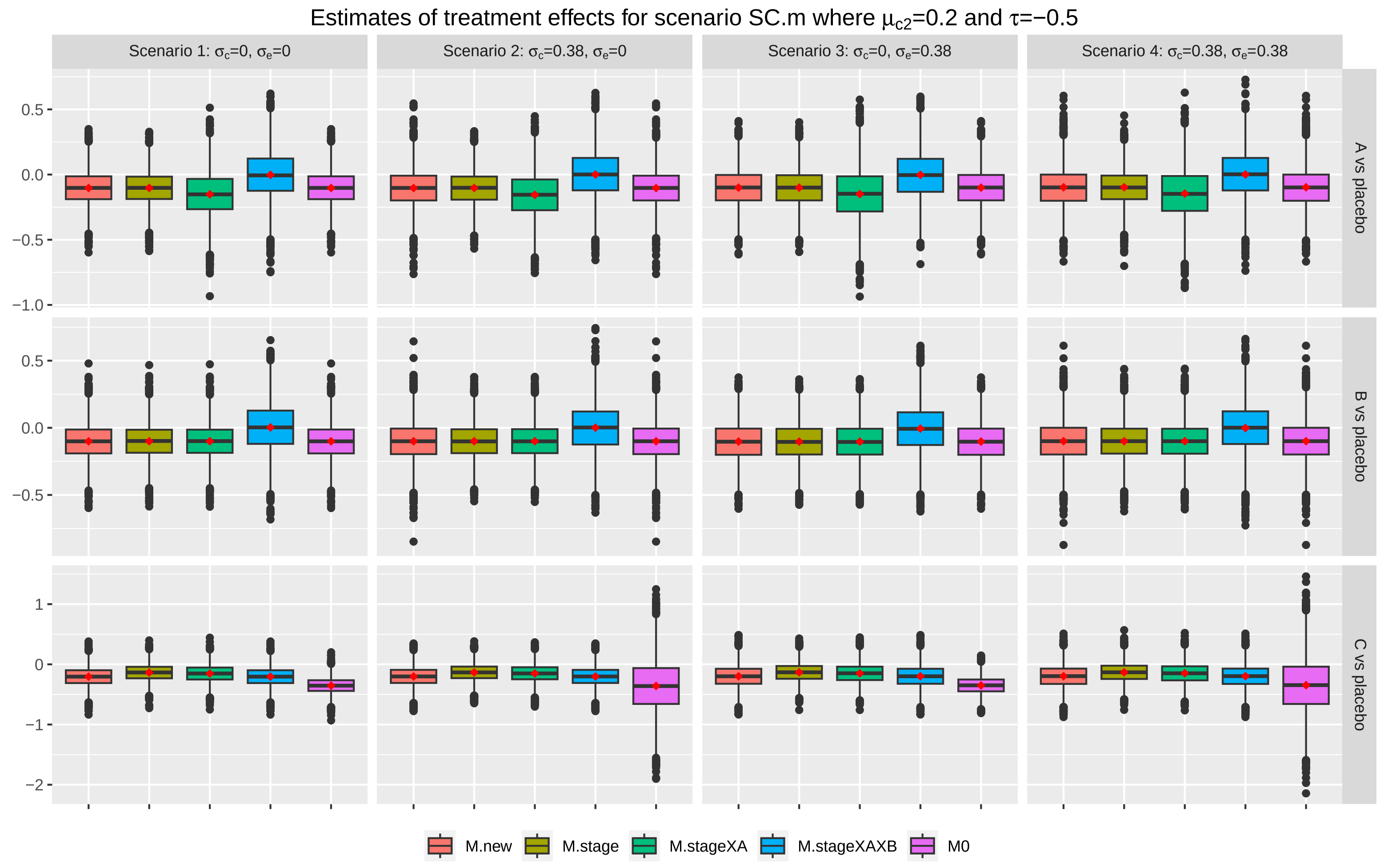

Figure 2: Box plots of sample estimates for each research comparison for scenario SC.m with $\tau = -0.5, \mu_{C2} = 0.2$ and other stage wise means are equal to 0. Arm C is added after recruitment of 50% of n_j, $j = 0, A, B$.

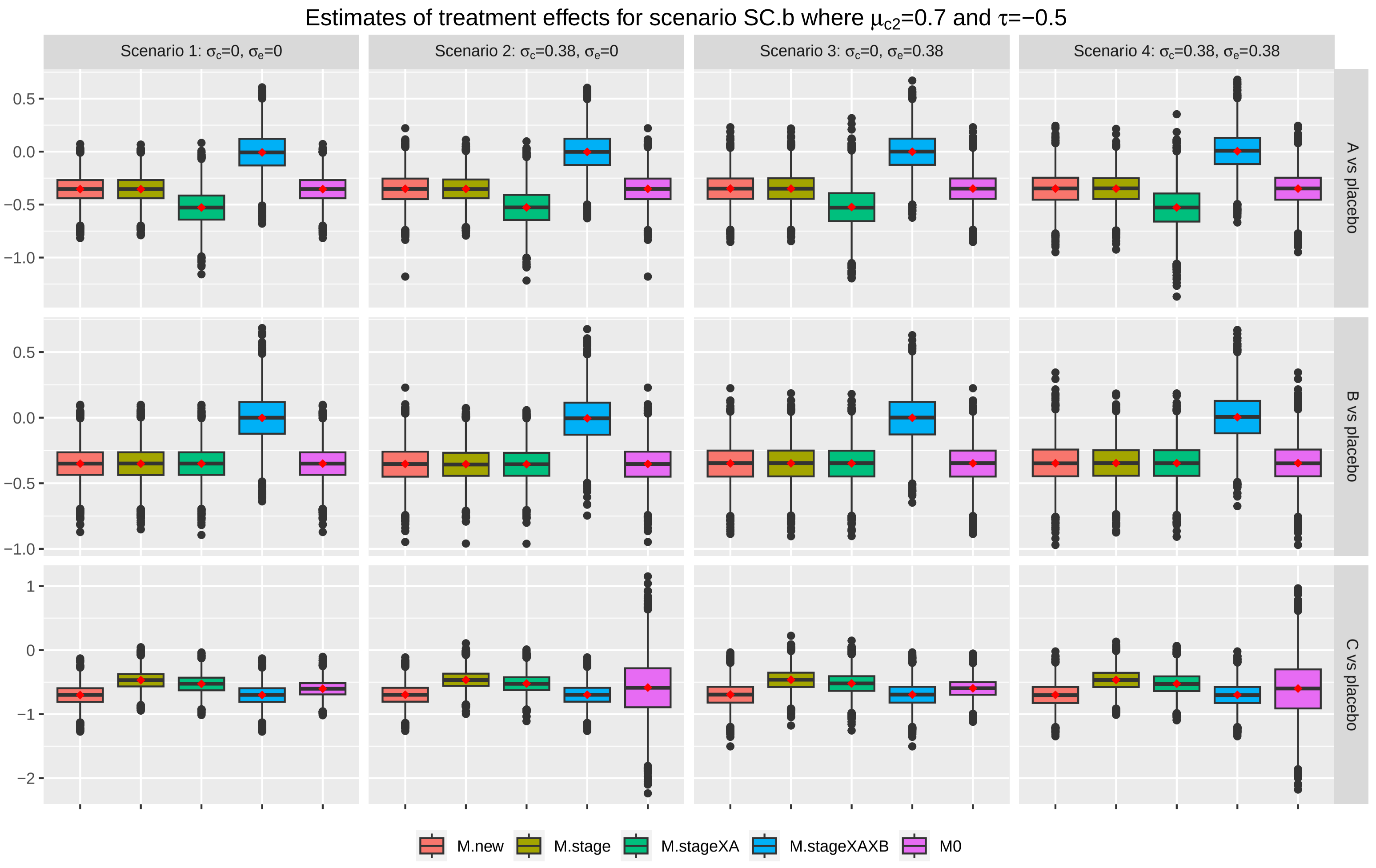

Figure 3: Box plots of sample estimates for each research comparison for scenario SC.b with $\tau = -0.5, \mu_{C2} = 0.7$ and other stage wise means are equal to 0. Arm C is added after recruitment of 50% of n_j, $j = 0, A, B$.

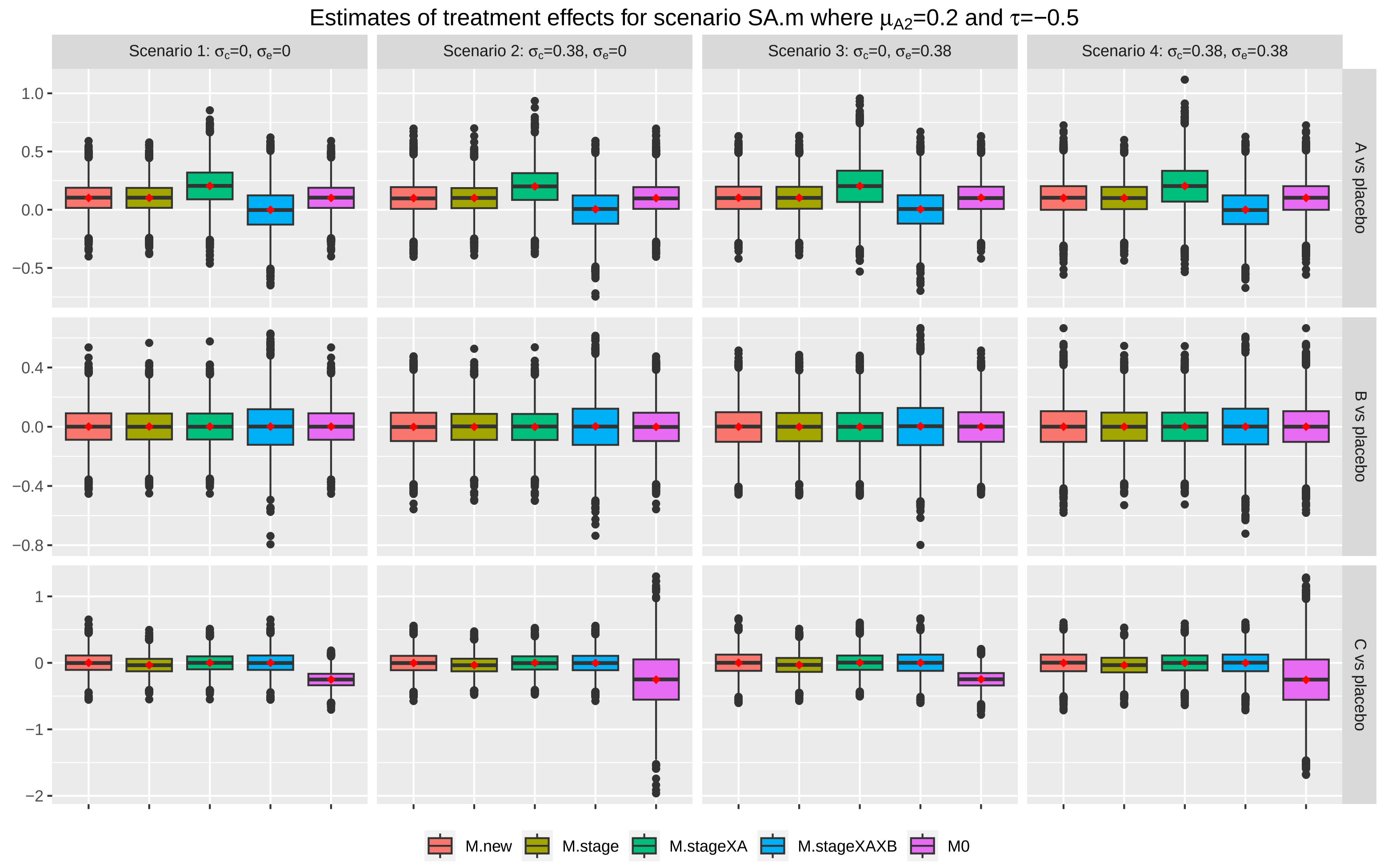

Figure 4: Box plots of sample estimates for each research comparison for scenario SA.m with $\tau = -0.5, \mu_{A2} = 0.2$ and other stage wise means are equal to 0. Arm C is added after recruitment of 50% of n_j, $j = 0, A, B$.

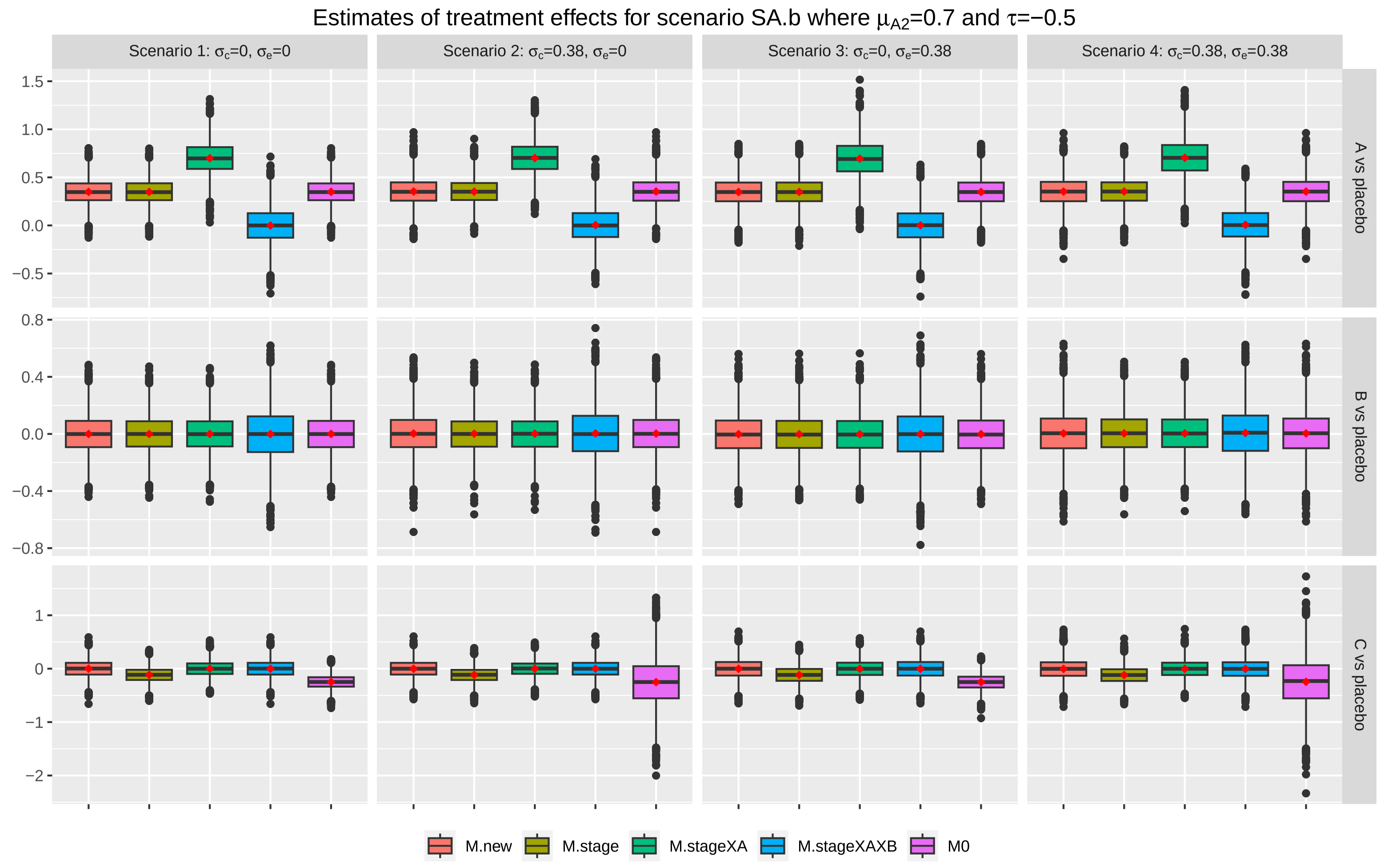

Figure 5: Box plots of sample estimates for each research comparison for scenario SA.m with $\tau = -0.5, \mu_{A2} = 0.7$ and other stage wise means are equal to 0. Arm C is added after recruitment of 50% of n_j, $j = 0, A, B$.

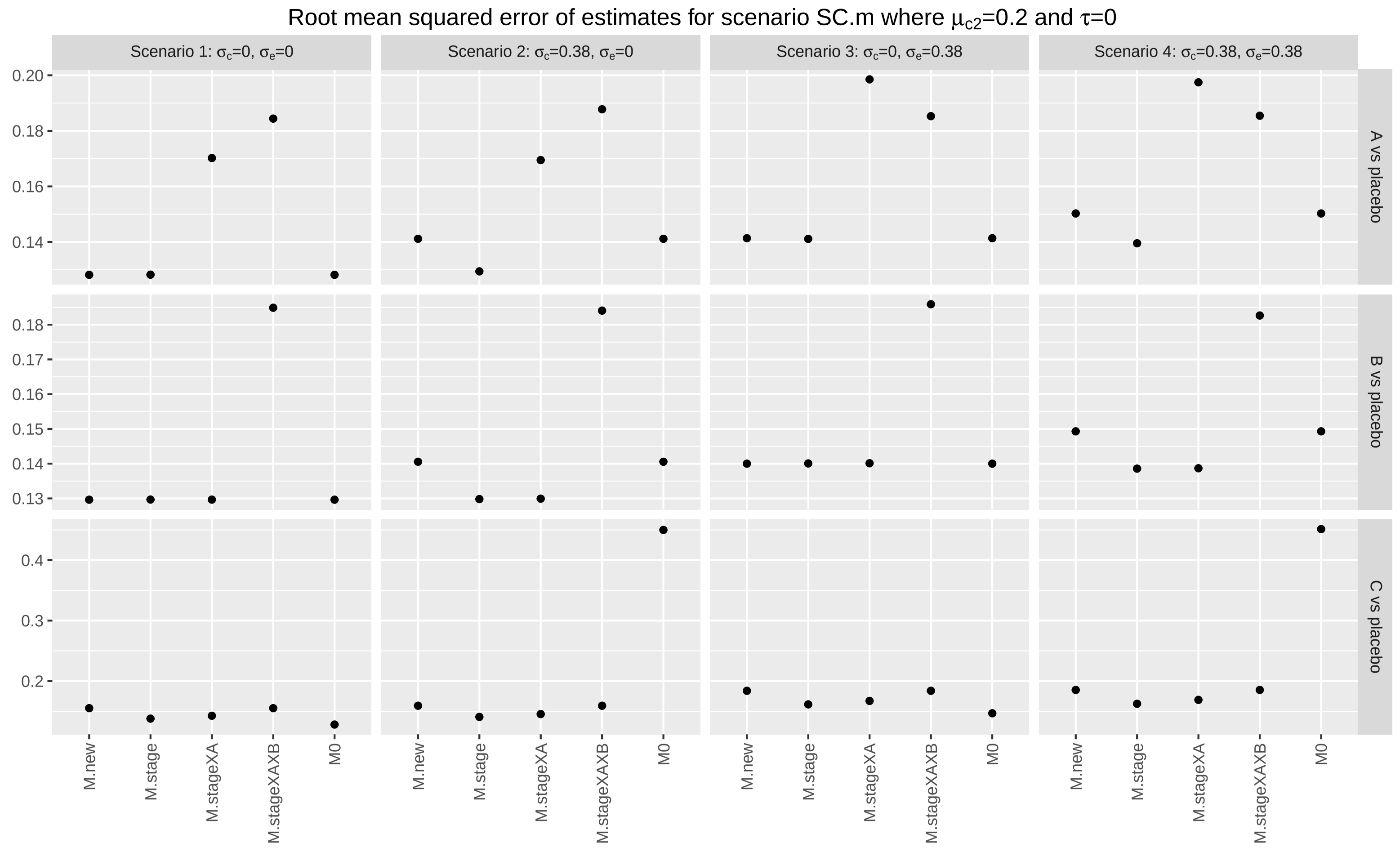

Figure 6: Root mean squared error of sample estimates for each research comparison for scenario SC.m and $\tau = 0$. Arm C is added after recruitment of 50% of n_j, $j = 0, A, B$.

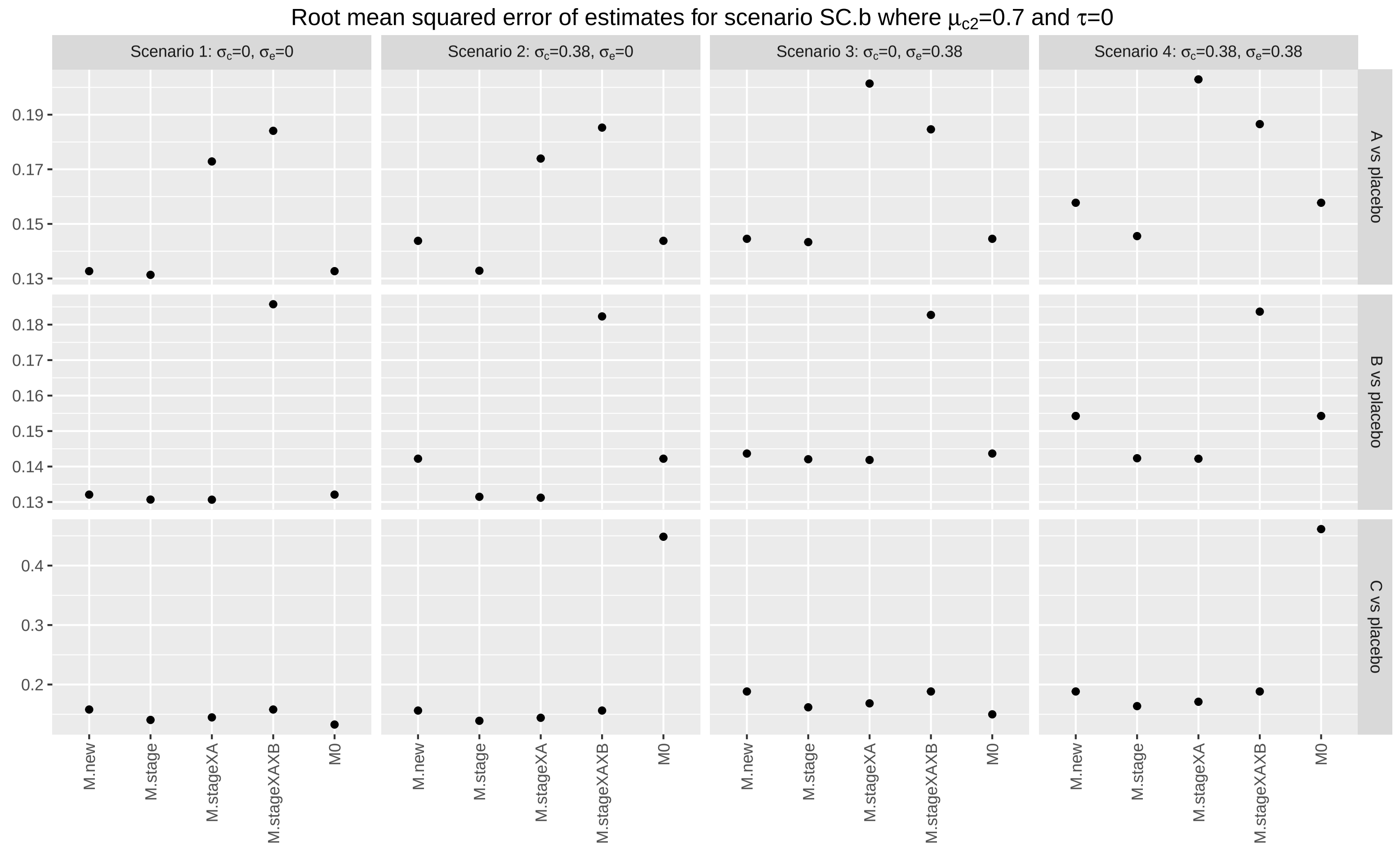

Figure 7: Root mean squared error of sample estimates for each research comparison for scenario SC.b and $\tau = 0$. Arm C is added after recruitment of 50% of n_j, $j = 0, A, B$.

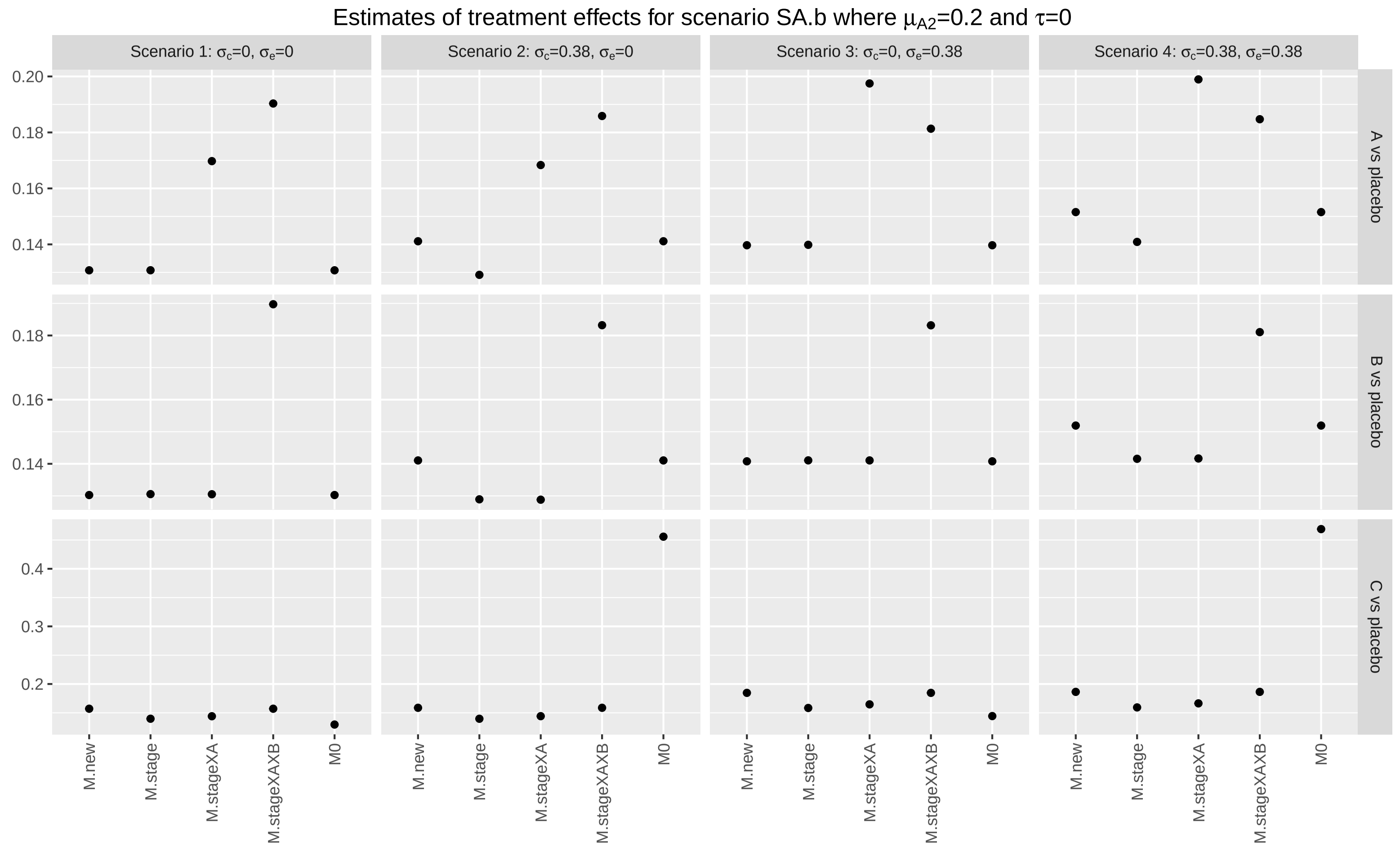

Figure 8: Root mean squared error of sample estimates for each research comparison for scenario SA.m and $\tau = 0$. Arm C is added after recruitment of 50% of n_j, $j = 0, A, B$.

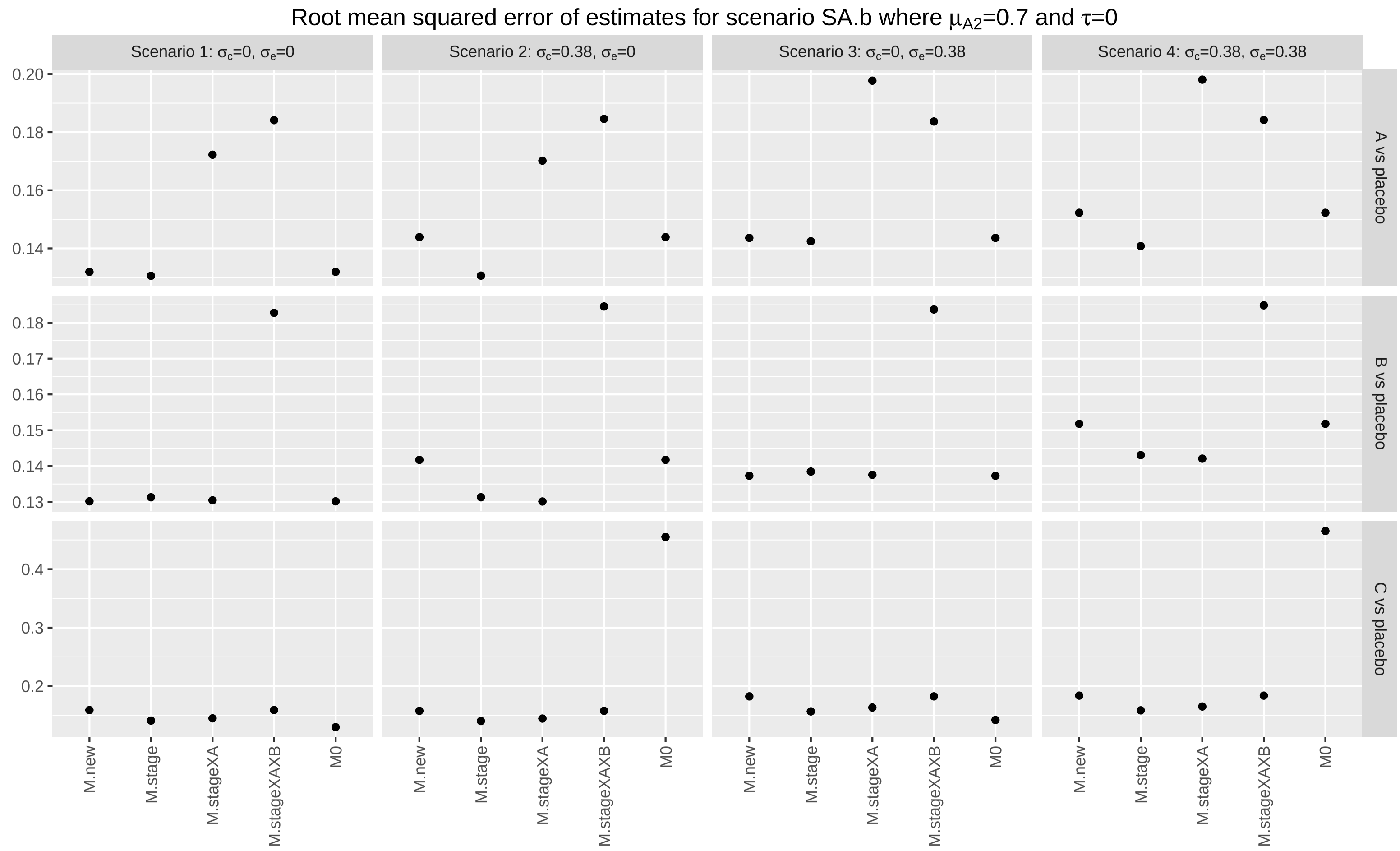

Figure 9: Root mean squared error of sample estimates for each research comparison for scenario SA.b and $\tau = 0$. Arm C is added after recruitment of 50% of n_j, $j = 0, A, B$.

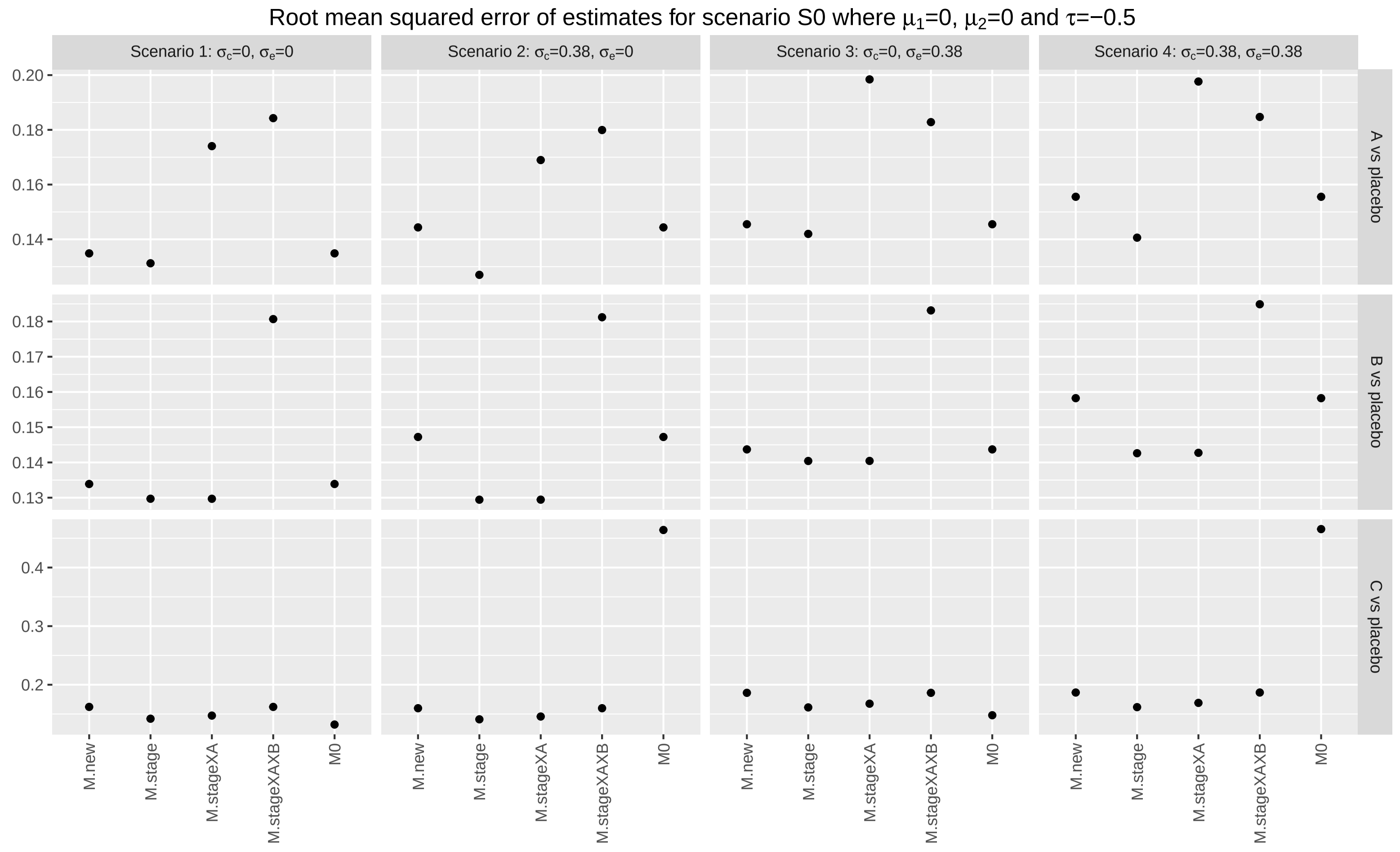

Figure 10: Root mean squared error of sample estimates for each research comparison for scenario S0 and $\tau = -0.5$. Arm C is added after recruitment of 50% of n_j, $j = 0, A, B$.

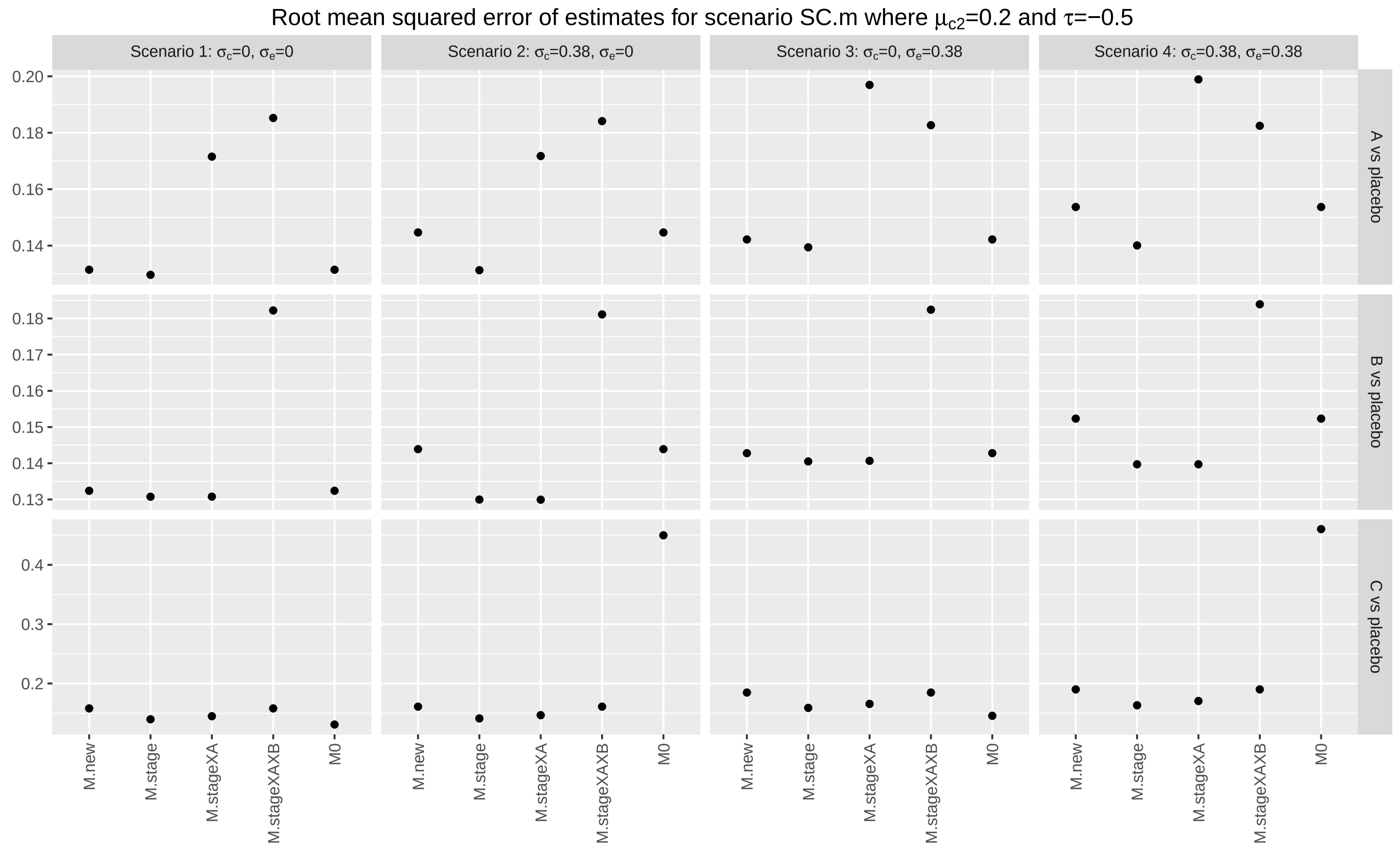

Figure 11: Root mean squared error of sample estimates for each research comparison for scenario SC.m and $\tau = -0.5$. Arm C is added after recruitment of 50% of n_j, $j = 0, A, B$.

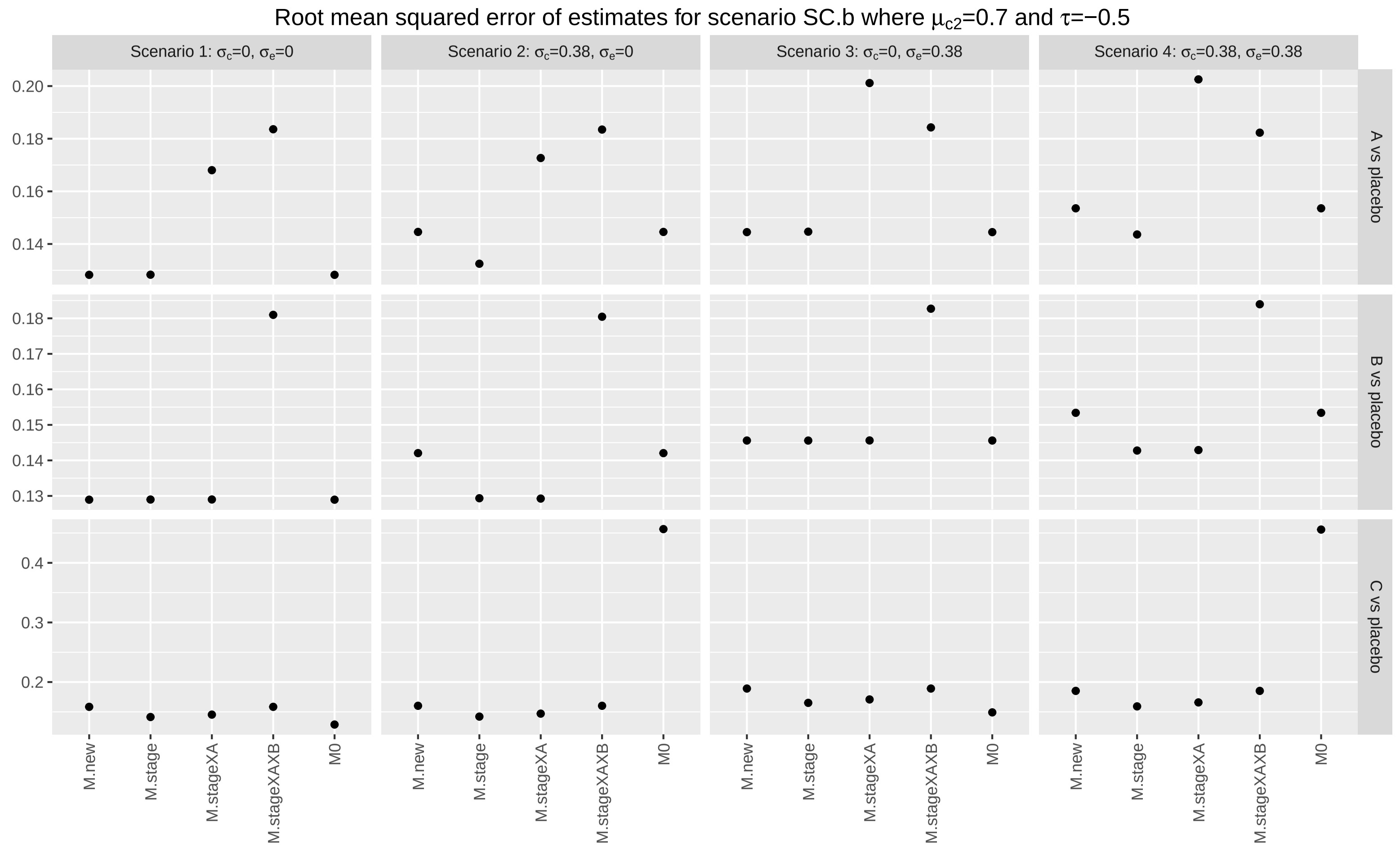

Figure 12: Root mean squared error of sample estimates for each research comparison for scenario SC.b and $\tau = -0.5$. Arm C is added after recruitment of 50% of n_j, $j = 0, A, B$.

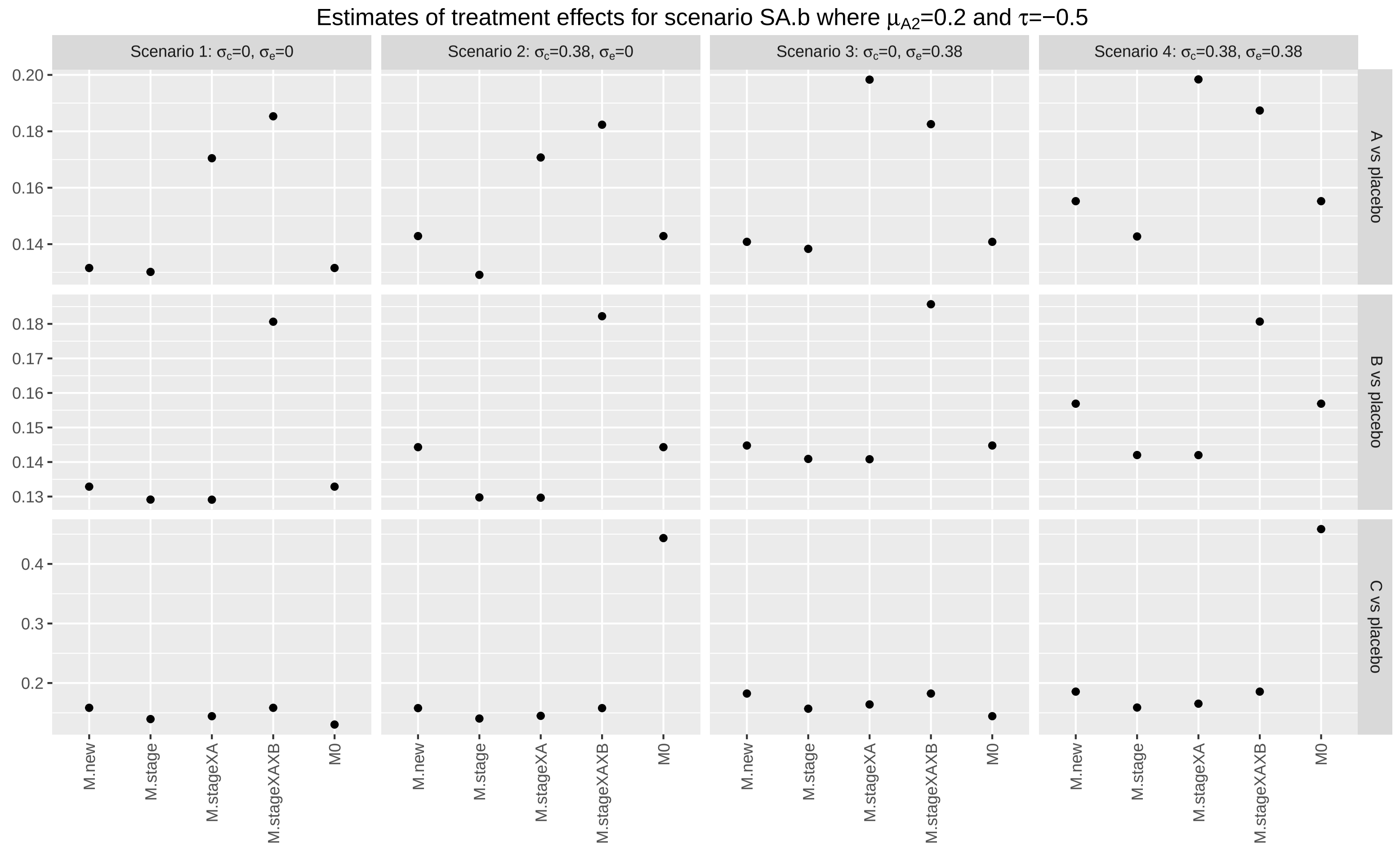

Figure 13: Root mean squared error of sample estimates for each research comparison for scenario SA.m and $\tau = -0.5$. Arm C is added after recruitment of 50% of n_j, $j = 0, A, B$.

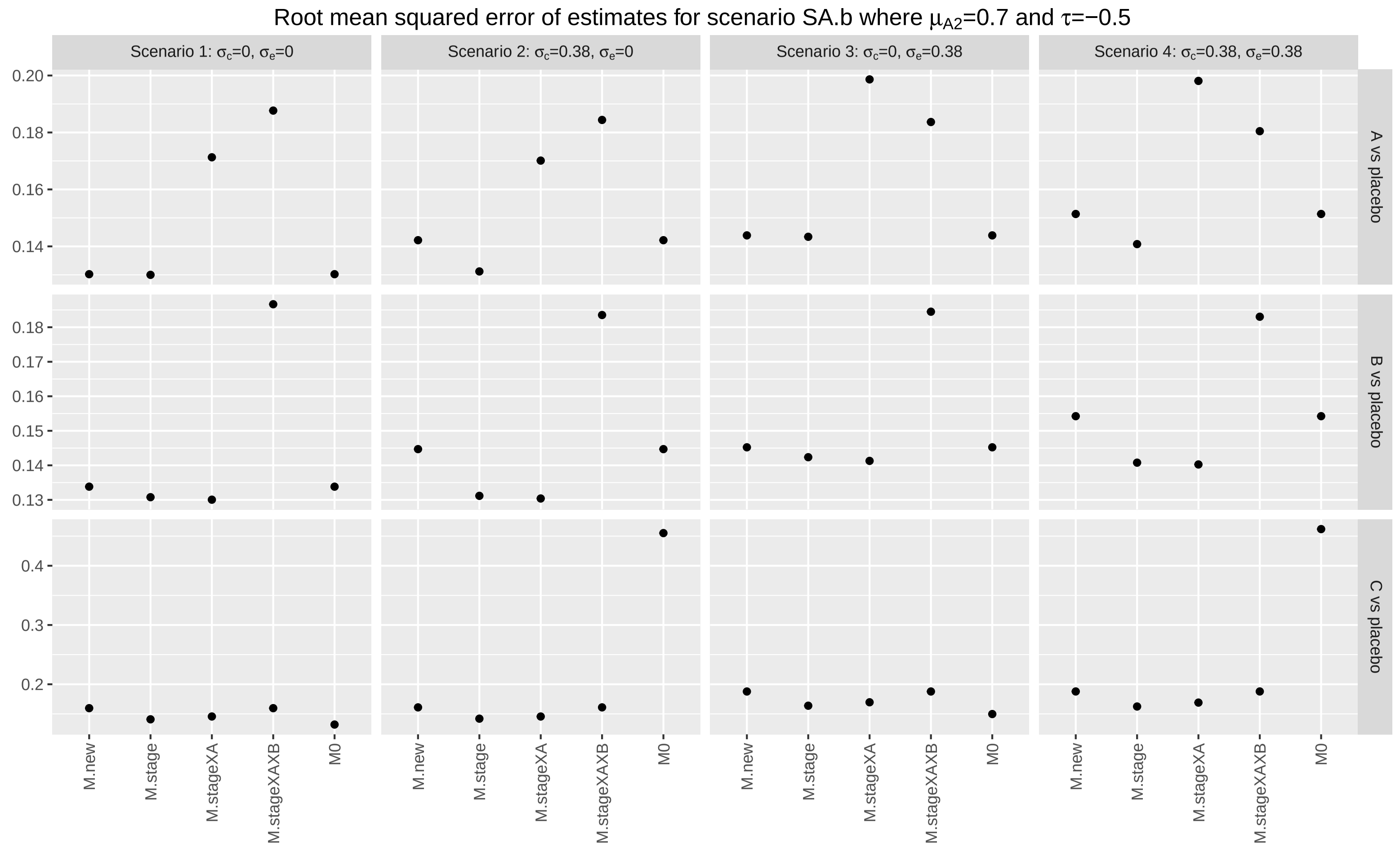

Figure 14: Root mean squared error of sample estimates for each research comparison for scenario SA.b and $\tau = -0.5$. Arm C is added after recruitment of 50% of n_j, $j = 0, A, B$.
